# New Tin (IV) and Organotin (IV) Complexes with a Hybrid Thiosemicarbazone/Hydrazone Ligand: Synthesis, Crystal Structure, and Antiproliferative Activity

**DOI:** 10.1155/2024/1018375

**Published:** 2024-04-03

**Authors:** Belén Blázquez-Tapias, Satyajit Halder, M. Antonia Mendiola, Nivedita Roy, Nilima Sahu, Chittaranjan Sinha, Kuladip Jana, Elena López-Torres

**Affiliations:** ^1^Departamento de Química Inorgánica, Universidad Autónoma de Madrid, Cantoblanco, Madrid 28049, Spain; ^2^Division of Molecular Medicine, Bose Institute, Kolkata 700 056, India; ^3^Department of Chemistry, Jadavpur University, Kolkata 700 032, India

## Abstract

Nowadays, the search for new chemotherapeutic agents with low toxicity and high selectivity is a major concern. In this paper, we report the synthesis and characterization of a hybrid thiosemicarbazone/hydrazone ligand in its neutral form (L^1^H_2_) and as the chloride salt ([L^1^H_3_]Cl)-, three diorganotin (IV) complexes, and one complex with Sn (IV). The compounds have been fully characterized by IR, mass spectra, ^1^H, ^13^C, and ^119^Sn NMR, ^119^Sn CP/MAS NMR, and by single crystal X-ray diffraction. The organotin compounds have the empirical formula [SnR_2_L^1^] (*R* = Me, Bu, and Ph), but in the solid state, they are polymeric species with seven coordination number due to weak coordination of the pyridine nitrogen, whereas in solution, the polymeric structure is lost to afford hexacoordinate monomeric species. Reaction with SnI_4_ yields complex [Sn (L^1^)_2_]·EtOH, with the metal in a distorted dodecahedral arrangement. We have evaluated the antiproliferative activity of the two forms of the ligands and the four coordination compounds against MDA-MB-231, HeLa, PC3, and HepG2 cancer cell lines, and WI-38 normal cell line, and all the compounds present higher activity than cisplatin, used as the standard control. To investigate the mode of action, we have selected the most active complex, containing phenyl substituents, and used the triple negative breast cancer cell line MDA-MB-231. The results show that the complex induces apoptotic cell death promoted by generation of reactive oxygen species and by disruption of mitochondrial membrane potential.

## 1. Introduction

Cancer is one of the most prevalent diseases causing 30% of the premature deaths in adults between 30 and 70 years, and it is estimated that in 2040, it will be responsible for 20 million deaths. Therefore, the development of new chemotherapeutic agents is a big concern. Since it was licensed in 1979 for clinical use, cisplatin remains as the most used metallodrug in the treatment of cancer, being successful in the front-line treatment for ovarian, testicular, breast, and kidney cancers, but less effective against lung cancer and other common tumors. In addition, cisplatin presents severe side-effects such as nephrotoxicity and several types of cancer have developed resistance. Hence, cisplatin and its analogues carboplatin, oxaliplatin, nedaplatin, and lobaplatin display important limitations, and during the last decades, there has been an extensive search of complexes with other metals that present higher selectivity and lower toxicity [1–6].

Since Gielen and coworkers reported the antitumor activity of organotin (IV) compounds [7, 8], they have attracted a great attention as potential metallodrugs [9–12]. Reaction of di- and triorganotin (IV) species with a wide range of ligands leads to the synthesis of a number of complexes with cytotoxic activity against several solid and hematological cancers that, in many cases, display higher antiproliferative activity and better excretion profiles, together with lower toxicity and fewer side effects than platinum-based drugs [13–15]. This activity strongly depends on the number and nature of the organic groups directly linked to the tin atom since they modulate the lipophilicity and, therefore, the ability to penetrate the cell membrane. Usually, the organic substituents, *R*, are alkyl groups such as methyl, ethyl, or butyl or aryl groups such as phenyl. From the data reported in the literature, it seems that the highest activity is found with [SnR_3_]^+^ derivatives followed by [SnR_2_]^2+^ [16–18], although there are some [SnPh_2_]^2+^ complexes that present higher activity than [SnPh_3_]^+^ analogues, since they can intercalate with DNA more effectively [19]. In addition, the nature of these groups also affects the cytotoxicity [20, 21] and, for example, for aryl derivatives, the activity decreases when the steric demand increases. The organotin (IV) compounds, as other metal complexes, can effectively induce apoptosis and generate reactive oxygen species which create oxidative stress in cancerous cells [22–25].

Thiosemicarbazones are a well-known class of ligands which have proved to present a wide range of pharmacological applications, both in diagnosis (as radiopharmaceuticals for PET [26–28] and SPECT [29–31]) and therapy since the free ligands and their complexes possess, among others, antiviral [32, 33], antimicrobial [34, 35], antitumor [36, 37], or antioxidant activity [38]. In terms of coordination chemistry, they are very versatile ligands because they have hard and soft donor atoms and an acidic NH group that can be deprotonated. In addition, they form very stable coordination complexes due to the formation of a five-member chelate ring, so they are ideal candidates for the development of new anticancer drugs.

For pharmacological applications, stability is a mandatory characteristic of the compounds, so ligands with high denticity are frequently used. Since the organotin (IV) moiety is considered hard and using our expertise in the synthesis of dissymmetric thiosemicarbazone ligands [39–41], we have synthesized a tetradentate hybrid thiosemicarbazone/hydrazone ligand with a N_2_OS donor set (both as a neutral molecule and as a chloride salt), which forms stable complexes with [SnR_2_]^2+^ derivatives (*R* = Me, Bu, and Ph) as well as with SnI_4_, leading to the formation of four new coordination compounds. We have studied their cytotoxicity against several cancer cell lines, namely, MDA-MB-231, HeLa, PC3, and HepG2 and one human normal lung fibroblast cell line, WI-38. For the most active one, we have performed several *in vitro* experiments to assess its mode of action. As far as we know, these investigations on organotin (IV) complexes with this type of tetradentate hybrid thiosemicarbazone/hydrazone ligands are not reported to date.

## 2. Experimental

### 2.1. Materials and Methods

Chemicals and reagents were used as received and were obtained from ABCR (Germany), Merck (India), Himedia (India), Invitrogen (India), SRL (India), and Sigma-Aldrich (USA). DCFDA (dichlorodihydrofluorescein diacetate) (#D6883) was purchased from Sigma-Aldrich (India). Foetal bovine serum (#16000044) was obtained from Gibco (USA) and MEM (minimum essential medium), sodium pyruvate, MEM nonessential amino acids, L-glutamine, and gentamicin were procured from Hi-Media (India). Microanalyses were registered in a LECO CHNS-932 Elemental Analyzer. IR spectra in KBr pellets were acquired on a Jasco FT/IR-410 spectrophotometer in the 4000−400 cm^−1^ range. Molar conductivity was measured using a freshly prepared DMF solution (ca. 10^−3^ M) at 25°C with a Crison EC-Meter BASIC 30 + instrument. The ESI mass spectra were obtained on a Q-STAR PULSAR I instrument using a hybrid analyzer QTOF (quadrupole time-of-flight). ^1^H, ^13^C{^1^H} and ^119^Sn{^1^H} NMR spectra were recorded on a spectrometer Bruker AVIII HD-300 MHz using DMSO-d_6_ as solvent and TMS (^1^H and ^13^C{^1^H}) or SnMe_4_ (^119^Sn{^1^H}) as the internal reference. *J* values are given in Hz. ^119^Sn{^1^H} CP/MAS NMR spectra were recorded at 298 K in a Bruker AV400WB spectrometer equipped with a 4 mm MAS (magic‐angle spinning) NMR probe was obtained using a cross‐polarization pulse sequence using spinning rates of 10–14 KHz, pulse delays of 30 s, contact times of 8 ms, and two‐pulse phase-modulated high power proton decoupling. Chemical shifts are reported relative to SnMe_4_, using tin (IV) oxide as a secondary reference.

### 2.2. Synthesis of the Organic Molecules

The atom labeling used for spectra assignments is in Supplementary Information (S1).

#### 2.2.1. Diacetyl-2-Thiosemicarbazone, HATs

The following compound was obtained following the procedure previously described (Reference [42]): *ν*_max_/cm^−1^ 3399, 3326 and 3182 (NH), 1685 (CO), 1587 (NH_2_), 852 (thioamide IV). *δ*H(300 MHz, DMSO-d_6_, Me_4_Si) 10.58 (1 H, s, NH), 8.71 (1 H, s, NH_2_), 8.10 (1 H, s, NH_2_), 2.40 (3 H, s, CH_3_CO), 1.97 (3 H, s, CH_3_CN).

#### 2.2.2. Diacetyl-2-(Thiosemicarbazone)-3-(Isonicotinichydrazonium) Chloride, [L^1^H_3_]Cl

To a suspension of HATs (0.250 g, 1.58 mmol) in 18 mL of absolute ethanol with 6 drops of conc. HCl, a solution of isonicotinic acid hydrazide (0.217 g, 1.58 mmol) in 2 mL of water, 1 ml of ethanol, and two drops of HCl were added dropwise. The mixture was stirred for 2 h at room temperature. The yellow solid formed was filtered off, washed with ethanol, and dried in vacuum (0.492 g, 99%). Found: C, 41.75; H, 5.02; N, 26.54; S, 10.30. C_11_H_15_N_6_OSCl (MW 314.76 gmol^−1^) requires C, 41.97; H, 4.80; N, 26.70; S, 10.19. *ν*_max_/cm^−1^ 3442, 3301 and 3152 (NH), 2557 (SH), 1671 (CO), 1634 (CN), 1606 (NH_2_), 1542 (thioamide I + amide II), 1385 (NH_2_), 876 (thioamide IV). *δ*H(300 MHz, DMSO-d_6_, Me_4_Si) 11.67 (1 H, s, H_2_), 11.38 (1 H, s, H_2_), 10.34 (1 H, s, H_5_), 10.29 (1 H, s, H_5_), 8.98 (4 H, dd, *J* = 6.4, *J* = 1.5, py), 8.47 (1 H, br s, H_1a_), 8.43 (1 H, br s, H_1a_), 8.23 (2 H, d, *J* = 6.5, py), 8.14−8.09 (2 H, m, py), 7.93 (1 H, br s, H_1b_), 7.87 (1 H, br s, H_1b_), 6.79 (2 H, br s, NH-py), 2.30 (6 H, s, CH_3_), 2.21 (6 H, s, CH_3_).

#### 2.2.3. Diacetyl-2-(Thiosemicarbazone)-3-(Isonicotinichydrazone) L^1^H_2_

A suspension of [L^1^H_3_]Cl (0.600 g, 1.91 mmol) and lithium hydroxide monohydrate (0.080 g, 1.91 mmol) in 12 mL of absolute ethanol was stirred under reflux for 24 h. The white solid formed was filtered off, washed with ethanol, and dried in vacuum (0.493 g, 93%). Found: C, 47.25; H, 5.18; N, 30.03; S, 11.43. C_11_H_14_N_6_OS (MW 278.31 gmol^−1^) requires C, 47.47; H, 5.07; N, 30.20; S, 11.52. *ν*_max_/cm^−1^ 3369, 3261 and 3169 (NH), 1670 and 1655 (CO), 1626 (CN), 1603 (NH_2_), 1548 (thioamide I + amide II), 1367 (NH_2_), 851 (thioamide IV). *δ*H(300 MHz, DMSO-d_6_, Me_4_Si) 11.01 (1 H, br s, H_2_), 10.34 (1 H, br s, H_5_), 8.75 (2 H, br s, H_7_ + H_8_), 8.45 (1 H, s, H_1a_), 7.90 (1 H, s, H_1b_), 7.78 (2 H, br s, H_6_ + H_9_), 2.26 (3 H, s, CH_3_), 2.21 (3 H, s, CH_3_). *δ*C{^1^H} (75 MHz, DMSO-d_6_, Me_4_Si) 179.5 (C_1_), 163.4 (C_4_), 156.0, 148.7 (C_2_, C_3_,), 150.5 (C_7_ + C_8_), 141.9 (C_5_), 122.4 (C_6_ + C_9_), 12.6, 11.9 (C_10_, C_11_).

### 2.3. Synthesis of the Tin Compounds

#### 2.3.1. [SnMe_2_L^1^]_n_, 1

To a hot suspension of [L^1^H_3_]Cl (0.100 g, 0.318 mmol) and lithium hydroxide monohydrate (0.040 g, 0.953 mmol) in 12 mL of absolute ethanol, a solution of SnMe_2_Cl_2_ (0.073 g, 0.318 mmol) in 2 mL of the same solvent was added and the mixture was stirred under reflux for 2 h. The orange solid formed was filtered off, washed with ethanol, and dried in vacuum (0.112 g, 83%). Found: C, 36.55; H, 4.60; N, 19.58; S, 7.42; SnC_13_H_18_N_6_OS (MW 425.07 gmol^−1^) requires C, 36.76; H, 4.27; N, 19.77; S, 7.55. *ν*_max_/cm^−1^ 3282 and 3168 (NH), 1671 (CO), 1608 (NH_2_), 1561 (CN), 1523 (thioamide I + amide II), 1368 (NH_2_), 816 (thioamide IV). *δ*H(300 MHz, DMSO-d_6_, Me_4_Si) 8.70 (2 H, dd, *J* = 6.0, *J* = 1.6, H_7_ + H_8_), 7.95 (2 H, dd, *J* = 6.0, *J* = 1.6, H_6_ + H_9_), 6.98 (2 H, br s, H_1a_ + H_1b_), 2.50 (3 H, s, CH_3_), 2.38 (3 H, s, CH_3_), 0.57 (6 H, s, SnMe_2_, *J*_Sn-H_ = 112). *δ*C{^1^H}(75 MHz, DMSO-d_6_, Me_4_Si) 178.9 (C_1_), 168.1 (C_4_), 151.8, 150.3, 146.5 (C_2_, C_3_, C_7_, C_8_), 144.2 (C_5_) 122.2 (C_6_ + C_9_), 16.0, 15.2 (C_10_, C_11_). *δ*^119^Sn{^1^H}(111 MHz, DMSO-d_6_, Me_4_Sn) −241.4. *δ*^119^Sn{^1^H} CP/MAS (149 MHz, Me_4_Sn) −416. m/z 427.04 [M + H] ^+^, 74%. Λ_M_ (Ω^−1^ cm^2^·mol^−1^, DMF): 2.3.

#### 2.3.2. [SnBu_2_L^1^]_n_, 2

This complex was obtained following the same procedure described for the synthesis of **1** but adding SnBu_2_Cl_2_ (0.100 g, 0.318 mmol) to yield an orange solid (0.110 g, 68%). Found: C, 44.92; H, 6.15; N, 16.30; S, 6.44; SnC_19_H_30_N_6_OS (MW 509.22 gmol^−1^) requires C, 44.81; H, 5.94; N, 16.50; S, 6.30. *ν*_max_/cm^−1^ 3395 (NH), 1632 (CO), 1604 (NH_2_), 1572 (CN), 1523 (thioamide I + amide II), 1368 (NH_2_), 814 (thioamide IV). *δ*H(300 MHz, DMSO-d_6_, Me_4_Si) 8.65 (2 H, dd, *J* = 6.0, *J* = 1.4, H_7_ + H_8_), 9.94 (2 H, dd, *J* = 6.0, *J* = 1.4, H_6_ + H_9_), 6.96 (s, 2 H, H_1a_ + H_1b_), 2.50 (3 H, s, CH_3_), 2.37 (3 H, s, CH_3_). 1.21−1.03 (12 H, m, CH_2_-CH_2_-CH_2_-CH_3_), 0.67 (6 H, t, *J* = 7.0, CH_2_-CH_2_-CH_2_-CH_3_). *δ*C{^1^H}(75 MHz, DMSO-d_6_, Me_4_Si) 179.9 (C_1_), 170.3 (C_4_), 152.0, 150.0, 149.7 (C_2_ + C_3_ + C_7_ + C_8_), 143.1 (C_5_), 122.2 (C_6_ + C_9_), 33.3 (CH_2_-CH_2_-CH_2_-CH_3_), 27.7 (CH_2_-CH_2_-CH_2_-CH_3_), 26.3 (CH_2_-CH_2_-CH_2_-CH_3_), 16.6, 15.7 (C_10_, C_11_), 13.6 (CH_2_-CH_2_-CH_2_-CH_3_). *δ*^119^Sn{^1^H}(111 MHz, DMSO-d_6_, Me_4_Sn) −243.9. *δ*^119^Sn{^1^H} CP/MAS (149 MHz, Me_4_Sn) −441. m/z 511.13 [M + H]^+^, 100%. Λ_M_ (Ω^−1^ cm^2^·mol^−1^, DMF): 4.7.

#### 2.3.3. [SnPh_2_L^1^]_n_, 3

This complex was obtained following the same procedure described for the synthesis of **1** but adding SnPh_2_Cl_2_ (0.105 mg, 0.318 mmol) to yield an orange solid (0.127 g, 73%). Found: C, 50.56; H, 4.28; N, 15.33; S, 5.72. SnC_23_H_22_N_6_OS (MW 549.20 gmol^−1^) requires C, 50.30; H, 4.04; N, 15.30; S, 5.84. *ν*_max_/cm^−1^ 3444 and 3376 (NH), 1602 (CO + NH_2_), 1576 (CN), 1525 (thioamide I + amide II), 1377 (NH_2_), 816 (thioamide IV). *δ*H (300 MHz, DMSO-d_6_, Me_4_Si) 8.68 (2 H, dd, *J* = 6.0, *J* = 1.6, H_7_ + H_8_), 7.98 (2 H, dd, *J* = 6.0, *J* = 1.6, H_6_ + H_9_), 7.52 (4 H, dd, *J* = 7.7, *J* = 2.0, o-Ph), 7.23 (2 H, s, H_1a_ + H_1b_), 7.16−7.12 (6 H, m, *m*-Ph + *p*-Ph), 2.46 (3 H, s, CH_3_), 2.34 (3 H, s, CH_3_). *δ*^119^Sn{^1^H} (111 MHz, DMSO-d_6_, Me_4_Sn) −285.9. *δ*^119^Sn{^1^H} CP/MAS (149 MHz, Me_4_Sn) −489. m/z 573 [M + Na]^+^, 6%, 551.07 [M + H]^+^, 100%, 473.02 [(M − Ph, 50]^+^. Λ_M_ (Ω^−1^ cm^2^ mol^−1^, DMF): 3.2.

Complexes **1**–**3** were also obtained by reaction with L^1^H_2_ in the presence of two equivalents of LiOH. H_2_O with similar yields and purity.

#### 2.3.4. [Sn(L^1^)_2_]·EtOH, 4

To a suspension of [L^1^H_3_]Cl (0.100 g, 0.318 mmol) and lithium hydroxide monohydrate (0.040 g, 0.953 mmol) in 12 mL of absolute ethanol, a solution of SnI_4_ (0.099 g, 0.158 mmol) in 2 mL of the same solvent was added and the mixture was stirred under reflux for 2 h. The orange single crystals formed were filtered off, washed with ethanol, and dried in vacuum (0.097 g, 91%). Found: C, 40.46; H, 4.32; N, 23.14; S, 16.43; SnC_24_H_30_N_12_O_3_S_2_ (MW 717.36 gmol^−1^) requires C, 40.18; H, 4.21; N, 23.43; S, 16.55. *ν*_max_/cm^−1^ 3283 (NH), 1616 (CO), 1602 (NH_2_), 1571 (CN), 1538 (thioamide I + amide II), 1382 (NH_2_), 817 (thioamide IV). *δ*H(300 MHz, DMSO-d_6_, Me_4_Si) 8.61 (4 H, dd, *J* = 6.0, *J* = 1.6, H_7_ + H_8_), 7.70 (4 H, dd, *J* = 6.0, *J* = 1.6, H_6_ + H_9_), 7.54 (4 H, s, H_1a_ + H_1b_), 4.35 (1 H, t, *J* = 5.0, CH_3_CH_2_OH), 3.45 (2 H, qd, *J* = 7.0, *J* = 5.0, CH_3_CH_2_OH), 2.62 (6 H, s, CH_3_), 2.48 (3 H, s, CH_3_), 1.07 (3 H, t, *J* = 7.0 Hz, CH_3_CH_2_OH). *δ*C{^1^H}(75 MHz, DMSO-d_6_, Me_4_Si) 174.7 (C_1_), 167.3 (C_4_), 150.5 (C_7_ + C_8_), 149.9, 142.1, 141.5 (C_2_, C_3_, C_5_), 121,4 (C_6_ + C_9_), 16.0, 15.7 (C_10_, C_11_). *δ*^119^Sn{^1^H}(111 MHz, DMSO-d_6_, Me_4_Sn) −770.0. m/z 695.04 [(M + Na)^+^, 20%], 673.06 [(M + H)^+^, 100].

Reactivity of both ligands with SnR_3_Cl metal salts was also explored but in all the reaction conditions tested, the formation of the triazine resulting from the loss of the hydrazone branch and subsequent cyclization of the monothiosemicarbazone was observed, yielding the complexes that were previously reported by the group [42].

### 2.4. X-Ray Crystallography

Data for complexes **2** needle, **2** plate, and **4** were obtained with a Bruker Kappa Apex-II diffractometer containing an Apex-II CCD area detector operating with graphite monochromator (Mo K*α* radiation, *λ* = 0.71073 Å). Absorption corrections using multiple measurements of symmetry-equivalent reflections were carried out using SADABS [43]. The raw intensity data frames were integrated with the SAINT program that also corrected Lorentz and polarization effects [44]. The space groups were determined with WinGX and the structures were solved by direct methods using SHELXS-2018 [45]. Weighted *R* factors (*R*_*w*_) and all goodness of fit S are based on *F*^2^; conventional *R* factors (*R*) are based on *F* [46].

CCDC numbers 2259438, 2259439, and 2210005 for **2** needle, **2** plate, and **4**, respectively, contain the supplementary crystallographic data for this paper. These data can be obtained free of charge from the Cambridge Crystallographic Data Centre via https://www.cdcc.cam.ac.uk/data_request/cif.

### 2.5. Cell Culture and Maintenance

Four cancerous cell lines (human triple negative breast cancer cell line MDA-MB-231, human cervical cancer cell line HeLa, human prostate cancer cell line PC3, and human liver cancer cell line HepG2) and the human normal lung fibroblast cell line, WI-38, were obtained from the central cell repository of National Centre for Cell Science (NCCS), Pune. The cell lines were cultured in a T25 flask with Dulbecco's modified Eagle medium (DMEM), supplemented with 10% foetal bovine serum, nonessential amino acids, 1 mM sodium pyruvate, 2 mM L-glutamine, 100 mg/L streptomycin, 100 units/L penicillin, and 50 mg/L gentamycin in a 37°C humidified incubator containing 5% CO_2_.

### 2.6. MTT Cell Proliferation Assay

The MTT (3-(4,5-dimethylthiazolyl-2)-2,5-diphenyltetrazolium bromide) assay was performed in order to measure the cell proliferation rate and, conversely, the reduction in cell viability when treated with the ligands L^1^H_2_ and [L^1^H_3_]Cl and the tin compounds **1**–**4**. [47, 48] For these assays, cells were seeded on 96-well plates at a density of 5 × 10^3^ cells/well and preincubated for 24 hours. Primarily, the cells were then exposed to different concentrations of the compounds (0 *µ*M, 2.5 *µ*M, 5 *µ*M, 10 *µ*M, 20 *µ*M, and 40 *µ*M) for 24 h and cisplatin has been used as a standard control. As DMSO has been used to prepare the different concentrations of the compounds, we have added 0.5% DMSO in the control setup. After the incubation period, media was discarded from the 96-well plates and washed with 1 × PBS solution twice. Then, MTT (100 *µ*L; 0.5 mg/mL) was added to each well and incubated in a humidified incubator containing 5% CO_2_ at 37°C for 4 h. After discarding the supernatant, the purple-colored formazan crystals formed in the wells were dissolved with 100 *μ*L DMSO per well and the absorbance was estimated at 490 nm using a microplate reader. The experiments were repeated three times, and the cell viability was expressed as a percentage of the control experimental setup. After selecting the lead compound, MTT assay was performed again for compound **3** and cells were exposed to different concentrations (0 *µ*M, 0.25 *µ*M, 0.5 *µ*M, 1 *µ*M, 2 *µ*M, and 5 *µ*M) for 24 h. For all the MTT assays, three fully independent experiments have been performed, and each experiment has been conducted with biological triplicate samples.

### 2.7. Cell Cycle Profiling Assay by Propidium Iodide Staining

MDA-MB-231 cells were seeded at a density of 1 × 10^6^ cells/mL in each Petri dish for 24 h. Thereafter, the cells were treated with increasing concentration of complex **3** (0.25 *µ*M, 0.5 *µ*M, and 1 *µ*M) for 24 h and 0.5% DMSO was added in the control setup. Post treatment, cells were harvested into single cell suspension and fixed by 75% ethanol for 24 h. at −20°C. After centrifugation, the cells pellets were resuspended in 1 × PBS followed by RNaseA (20 *μ*M) treatment for 2 h at 37°C. Finally, propidium iodide was added and incubated at room temperature for 20 min. Subsequently, the samples were measured using BD FACSVerse flow cytometer (BD Biosciences, San Jose, CA) and analyzed using BD FACS DIVA software. CellQuest statistics was employed to quantitate the data at different phases of cell cycle, and histogram display of counts (*y* axis) versus DNA content (*x* axis, PI fluorescence) has been displayed [49].

### 2.8. Annexin V-FITC/PI Staining for Apoptosis Assay

Induction of apoptosis was quantified via flow cytometric analysis of control (0.5% DMSO control) and cells treated with complex **3** (0.25 *µ*M, 0.5 *µ*M, and 1 *µ*M) that were stained with annexin V-FITC/PI [50], using the Annexin V-FITC apoptosis detection kit according to the manufacturer's protocol (BD Bioscience). Post treatment cells were harvested with 1 × trypsin and washed in ice 1 × PBS followed by resuspension in 100 *µ*L of 1 × binding buffer solution supplied within the kit. Finally, cells were incubated with 5 *μ*L of annexin V-FITC and 5 *μ*L of PI for 15 min at room temperature in the dark before acquiring data using BD FACSVerse flow cytometer (BD Biosciences, San Jose, CA). Annexin V/FITC positive cells were regarded as apoptotic cells analyzed using Cell Quest software (BD Biosciences).

### 2.9. Measurement of Cellular ROS Using DCFDA

To measure the production of intercellular reactive oxygen species (ROS) produced by treatment with complex **3**, the DCFDA method was used [51]. MDA-MB-231 cells were seeded in a 6-well plate and treated with complex **3** (0.25 *µ*M, 0.5 *µ*M, and 1 *µ*M) for 24 h using arsenic as a positive control agent and NAC as negative control. Post treatment, the media was discarded and incubated with 10 *µ*M H_2_DCFDA for 30 min at 37°C. For fluorescence imaging, cells incubated with H_2_DCFDA were washed, resuspended in 1 × PBS, and directly imaged under a fluorescence microscope (Leica). For flow cytometric analysis, cells were then trypsinized, washed with 1 × PBS, and collected in centrifuge tubes. DCF fluorescence was measured using BD FACSVerse flow cytometer (BD Biosciences) and analyzed using Cell Quest software (BD Biosciences).

### 2.10. Detection of Mitochondrial Membrane Potential by JC1 Staining

The changes in mitochondrial membrane permeability were determined by JC1 (molecular probes) [52]. MDA-MB-231 cells were treated with compound **3** (0.25 *µ*M, 0.5 *µ*M, and 1 *µ*M) for the indicated time period, harvested, washed twice in 1 × PBS, resuspended in PBS supplemented with JC1 dye (**3** *µ*M final concentration), and incubated for 15 min in the dark at 37°C and flow cytometric analysis was immediately performed using a FACSVerse instrument (BD) or images were captured with fluorescent microscope (Leica).

### 2.11. Statistical Analysis

All the *in vitro* experiments have been performed thrice and the results were expressed as mean ± SEM. One-way ANOVA was followed by the Dunnett multiple comparison test. The level of significance was set at ^*∗∗∗*^(*P* < 0.001); ^*∗∗*^(*P* ≤ 0.01 − 0.001); and ^*∗*^(*P* ≤ 0.01 − 0.05) regarding control.

## 3. Results and Discussion

### 3.1. Synthesis

**Scheme 1:**
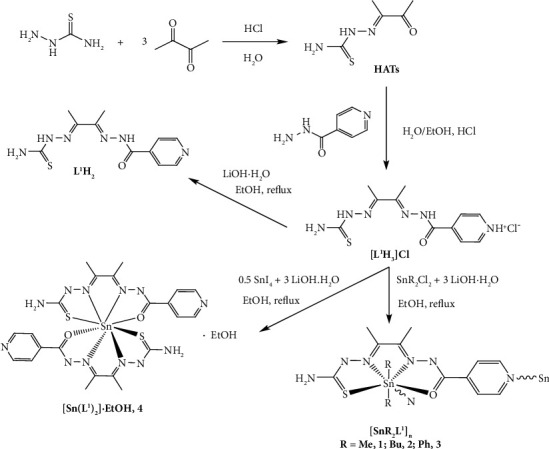
Synthesis of [L^1^H_3_]Cl, L^1^H_2_, and complexes **1**–**4**.

The synthesis of the dissymmetric ligand requires two successive steps as follows: First, the condensation reaction with thiosemicarbazide, followed by the addition of isonicotinic acid hydrazide. Both reactions need the presence of hydrochloric acid as catalyst that also leads to the protonation of the pyridine ring. Thus, the new ligand is isolated as the chloride salt [L^1^H_3_]Cl. Attempts to directly obtain the neutral ligand were unsuccessful since in the absence of hydrochloric acid, the dissymmetric ligand was not obtained. Nevertheless, the neutral ligand L^1^H_2_ can be synthesized by deprotonation of [L^1^H_3_]Cl with lithium hydroxide monohydrate (Scheme 1), but its reactivity is analogous to that of [L^1^H_3_]Cl, so the complexes were synthesized from [L^1^H_3_]Cl.

The reactivity of both ligands with the tin precursors has been explored (Scheme 1). In all the reactions, lithium hydroxide was added to induce ligand deprotonation. SnR_2_Cl_2_ (*R* = Me, Bu, and Ph) precursors afford complexes [SnR_2_L^1^]_n_ (**1**-**3**) in good yield, whereas the reaction with SnI_4_ leads to the formation of compound **4**, with the formula [Sn (L^1^)_2_]·EtOH.

Reactions with SnR_3_Cl (*R* = Me, Bu, and Ph) lead to the obtaining of the triazine derivatives that were previously published by the group from the reaction of diacetyl-2-thiosemicarbazone, HATs [42]. In these reactions, the hydrazone branch is lost and the NH_2_ group of the resulting monoketone reacts with the available carbonyl group to yield the 1,3,5-triazine-3-thione.

The spectroscopic and analytical data of the organometallic derivatives **1**-**3** indicate that all the compounds possess equivalent structures in the solid state as well as in solution. The elemental analyses for complexes **1**-**3** indicate a 1 : 1 metal to ligand ratio, whereas for complex **4**, it agrees with a 2 : 1 stoichiometry. In addition, the data show the absence of chloride or iodide, indicating the ligand acts as a dianionic donor.

The mass spectra of complexes **1**–**3** (S2–S4) confirm the 1 : 1:2 metal: ligand: R stoichiometry, showing the fragments [SnL^1^*R*_2_ + H]^+^ at 427.04, 511.13 and 551.07 uma for methyl, butyl, and phenyl derivatives and, in complex **3**, also the peak corresponding to [SnL^1^Ph]^+^. All of these peaks are consistent with the existence of monomeric species in solution. The 2 : 1 stoichiometry of complex **4** is confirmed by the peaks at 673.06 and 695.04 uma attributable to [Sn(L^1^)_2_ + H]^+^ and [Sn(L^1^)_2_ + Na]^+^, respectively, and the absence of peaks corresponding to the 1 : 1 stoichiometry (S5). For all the peaks, the experimental isotopic pattern agrees with the theoretical one.

### 3.2. Crystal Structures

The crystallographic and refinement data of the three compounds are summarized in S6 and selected bond distances are displayed in S7.

**Figure 1:**
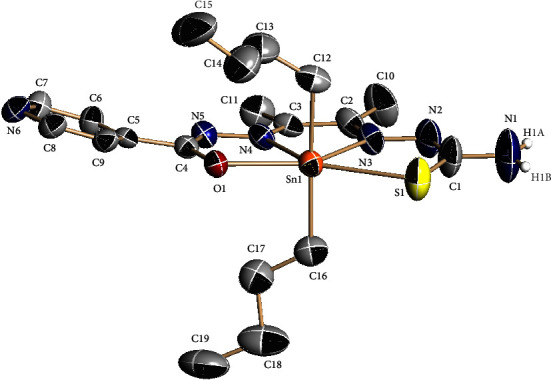
Asymmetric unit of complex **2** needle. Thermal ellipsoids at the 40% probability level. Hydrogen atoms, except those on nitrogen, are omitted for clarity.

**Figure 2:**
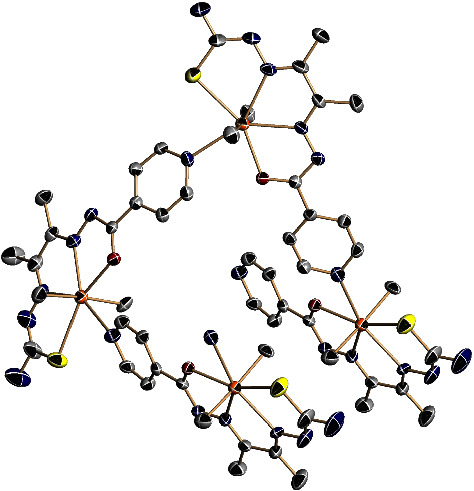
View of a part of the helical polymeric chain of complex **2** needle. Hydrogen atoms and three carbons on the butyl substituents have been omitted for clarity.

The following two different crystalline materials were isolated from a solution of complex **2** in DMSO-d_6_ + D_2_O 1 : 2: a big amount of very small needles and a small number of big plates. Both compounds could be satisfactorily analyzed by single crystal X-ray diffraction. The asymmetric unit of the needles contains [SnBu_2_L^1^] units (Figure 1) linked by a bond between the tin atom and the pyridine nitrogen of a neighbor molecule, leading to a helical 1D polymeric chain around the C_3_ axis, which is located on the *c* axis (Figure 2). This bond, with a Sn-N distance of 2.615(13) Å, is weak and, therefore, it is broken in DMF solution during the acquisition of the mass spectra, giving monomeric species. The ligand core is planar with a maximum deviation from the least-squares plane of 0.079 Å for C3. The pyridine ring is not coplanar with the ligand core and is forming an angle of 17.06°, probably due to the bond with the tin (IV) ion. The formation of the helical polymeric chain prevents the establishment of hydrogen bonds.

**Figure 3:**
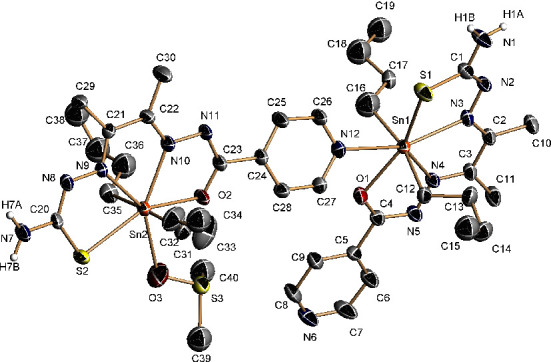
Molecular structure of complex **2** plate. Thermal ellipsoids at the 50% probability level. Hydrogen atoms (except those on N) and solvent molecules are omitted for clarity.

By contrast, the plates contain [SnBu_2_(DMSO)-*µ*-L^1^SnBu_2_L^1^] units, in which coordination of a dimethyl sulfoxide molecule to one of the tin atoms present in the asymmetric unit prevents the formation of the polymeric chain, resulting in the obtaining of dimers (Figure 3). In both structures (needle and plate), the ligand is acting as a N_2_SO tetradentate chelate donor and the nitrogen of the pyridine ring coordinates to another tin atom, forming the dimer (plate) or the polymer (needle). In the dimer, the Sn-pyridine bond is even longer than in the polymer, 2.6684(14) Å. Coordination of pyridine in only one of the ligands present in the asymmetric unit makes the ligands' skeletons to be different. In the one in which the pyridine is uncoordinated, the pyridine is coplanar with the rest of the ligand core except the sulfur atom, which is 0.611 Å under the plane. In the one in which the pyridine is coordinated, it is forming and angle of 9.39° with the ligand core (maximum deviation of 0.278 Å for S2). There are hydrogen bonds between the NH_2_ nitrogen N7 and the free pyridine nitrogen N6, forming tetramers that are linked by hydrogen bonds between N1 and the hydrazinic nitrogen N2, leading to the formation of chains running along the (0, 1, 1) direction (S8).

**Figure 4:**
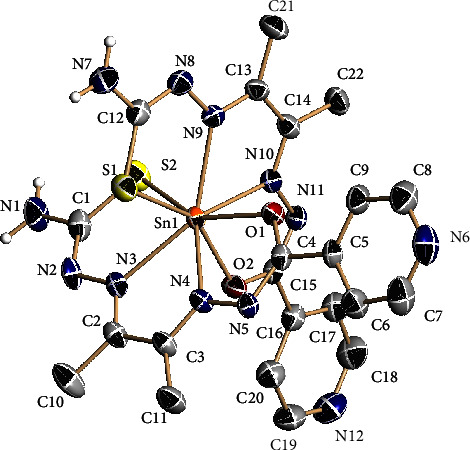
Molecular structure of complex **4**. Thermal ellipsoids at the 50% probability level. Hydrogen atoms and the ethanol molecule are omitted for clarity.

The molecular structure of complex **4** was also determined by single crystal X-ray diffraction (Figure 4) and consists of two doubly deprotonated ligands bound to the tin ion as N_2_SO tetradentate chelates, giving rise to the formation of six five-membered chelate rings that confers high stability to the complex. The tin atom is in a distorted dodecahedral environment, which is analogous to the structure of other tin (IV) complexes synthesized in the group [53, 54]. The presence of a crystallization ethanol molecule leads to the formation of intramolecular hydrogen bonds between the nitrogen atom of one of the pyridine rings and the OH group of the solvent molecule. Moreover, there exists a hydrogen bond between the OH of the ethanol and one of the NH_2_ groups, as well as between the NH_2_ and the pyridine that is not involved in hydrogen bonding with the ethanol, leading to the formation of chains in the (1,1,0) direction (S9). Bond distances in both ligands are remarkably similar and indicate some deal of electronic delocalization. The ligand's cores can be considered planar with maximum deviation from the least-squares plane of 0.2544 Å for S1 and 0.112 Å for S2. The main difference between both ligands is the orientation of the pyridine ring; in one of them, it is forming a dihedral angle of 21.55° with respect to the ligand skeleton, whereas in the other, the angle is 5.33°.

### 3.3. IR Spectroscopy

A table containing the most important bands observed in the IR spectra can be found in Supplementary Material (S10). The bands observed in the IR spectra of both forms of the ligand (S11 and S12) confirm the formation of the dissymmetric Schiff base. The spectrum of uncoordinated [L^1^H_3_]Cl shows a band at 2557 cm^−1^ corresponding to *ν*(S-H) that indicates the presence of the thiol tautomer. This band is very broad, probably due to the formation of hydrogen bonds. In the IR spectra of the complexes **1**–**3** (S13–S15), the bands corresponding to the organic groups attached to the tin ion are clearly observed, confirming the presence of the diorganotin (IV) moieties. The absence of these bands in the spectrum of complex **4** (S16) agrees with its structure. In complexes **1**–**4** can be observed a decrease in the number of *ν*(NH) bands, as expected after ligand deprotonation. The bands attributable to *ν*(C=N), *ν*(C=O), and *ν*(C=S) appear at different wavenumber than in the free ligand, which supports that the ligand behaves as a N_2_OS donor.

### 3.4. NMR Spectroscopy

^1^H, ^13^C{^1^H} and ^119^Sn{^1^H} NMR chemical shifts are listed in the experimental section and the spectra are collected in the Supplementary Material (S17–S30). The ^1^H NMR spectrum of [L^1^H_3_]Cl shows two sets of signals, suggesting a tautomeric thiol-thione equilibrium, supported by its IR spectrum. The spectrum of L^1^H_2_ shows all the signals corresponding to its structure, with the chemical shifts and the integrals expected. The hydrogen atoms H_1a_ and H_1b_ of the thiosemicarbazone arm are diastereotopic. However, in the spectra of complexes **1**–**3**, only one signal for both protons is observed. The signals corresponding to H_2_ and H_5_ have disappeared in the spectra of all the complexes due to its dianionic behavior. The signals belonging to the organic groups (Me, Bu, and Ph) bound to the tin can be observed in the spectra of the three complexes with a relationship of 2 : 1 with respect to the ligand. In the spectrum of complex **1**, the satellites corresponding to coupling with ^117^Sn and ^119^Sn can be clearly observed around the methyl signal. The substitution of ^2^*J*(^119^Sn-^1^H) = 98 Hz obtained from the ^1^H NMR spectrum of complex **1** in the corresponding Lockhart–Manders equation (55) *θ* = 0.0161[^2^*J*_(119Sn-1H)_]^2^−1.32[^2^*J*_(119Sn-1H)_] + 133.4 leads a value of 158.66° for the C-Sn-C angle in solution, which is in the range of 168.9 and 147.0° observed for similar complexes synthesized in the group and agrees with the methyl groups occupying the axial positions in a distorted octahedral arrangement [54] due to the breaking of the weak Sn-pyridine bond in solution. The spectrum of complex **4** shows a 1 : 2 ethanol: ligand ratio due to the crystallization ethanol molecule.

The ligand [L^1^H_3_]Cl and complex **3** are not soluble enough in common deuterated solvents to acquire a good ^13^C{^1^H} NMR spectrum. The ^13^C{^1^H} NMR spectrum of L^1^H_2_ shows all the signals expected for its structure. The spectra of complexes **1**, **2**, and **4** show that the signals attributable to C_2_ (C=N), C_3_ (C=N) and C_4_ (C=O) are shifted compared to free ligand, suggesting coordination of these groups to the metal. The signal of C_1_ (C=S) is almost at the same position as that in the free ligand although the sulfur atom is coordinated to the metal, but this is usually observed in thiosemicarbazone complexes due to electronic delocalization in the ligand backbone and *π*-back bonding.

^119^Sn NMR chemical shift is a useful tool to establish the chemical environment of the tin ion in a complex since it strongly depends both on the coordination number and the nature of the donor atoms bound to the metal ion. The values observed in the ^119^Sn{^1^H} NMR spectra in solution for complexes **1–3** are similar to other six-coordinate complexes with analogues environments [54], provided by one tetradentate ligand and two organic groups, indicating the breaking of the Sn-pyridine bond, whereas complex **4** agrees with a N_4_O_2_S_2_ coordination environment [53, 56, 57]. Since the chemical shifts in solution cannot be justified with the polymeric structure found for complex **2**, ^119^Sn{^1^H} CP/MAS NMR spectra of complexes **1**–**3** were also acquired (S31–S33), showing values of −416, −441, and −489 ppm, respectively, that clearly indicate a higher coordination number than in solution and correlates well with heptacoordinate structures. These experiments confirm that in the solid state, the three complexes present a polymeric structure that is lost in solution to form monomeric [SnL^1^*R*_2_] species, which are then responsible of the antitumor activity.

To analyze the stability of the monomeric species in water, we prepared a solution in a mixture containing DMSO-d_6_ + D_2_O 1 : 2 and registered the ^1^H NMR spectra after 24 h (S34–S37). Comparison with those obtained in pure DMSO-d_6_ shows that, as expected, the singlet around 7 ppm has disappeared, confirming that it corresponds to the NH_2_ group. The other signals appear at the same chemical shift and with the same intensity and multiplicity and no signals attributable to the free ligand are observed. This confirms that the ligand is not released in the presence of water and, therefore, a ligand exchange reaction does not occur, only the cleavage of the Sn-N bond and the loss of the polymeric structure to afford the monomeric hexa-coordinated species. This is also confirmed by the crystal structures of the two compounds isolated from the solution of complex **2**, which retain the [SnBu_2_L^1^] unit and do not present coordinated water molecules.

### 3.5. Cytotoxicity towards Several Cancer Cell Lines

**Figure 5:**
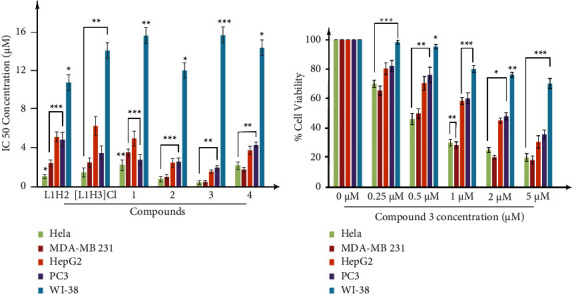
*In vitro* cytotoxicity measured by MTT. (a) IC_50_ in different cell lines. (b) Cytotoxic effect of complex **3** (0-5 *µ*M) in cell lines MDA-MB-231, HeLa, PC3, and HepG2 (cancer cells) and WI-38 (normal lung fibroblast cell line). The data have been repeated thrice and the results were expressed as the mean ± SEM. The level of significance was set at ^*∗∗∗*^*P* < 0.001; ^*∗∗*^*P* ≤ 0.01 − 0.001; and ^*∗*^*P* ≤ 0.01 − 0.05 in respect with the control.

To assess the ability of the compounds to block the proliferation of HeLa, MDA-MB-231, HepG2, and PC3 (cancer cells) and WI-38 (normal lung fibroblast cell line), MTT assay was performed using cisplatin as a standard control. Their antiproliferative efficacies in terms of IC_50_ (Figure 5(a) and Table 1) show that all the compounds possess significant cytotoxicity in the studied cancer cell lines, relative to the normal cell line WI-38 and exerted lower IC_50_ as compared to cisplatin. Remarkably, both ligands display high antiproliferative activity although it is slightly higher for L^1^H_2_ than for [L^1^H_3_]Cl in all the cell lines except PC3. It can also be observed that, in general, complexation increases cytotoxicity, mainly in the complexes bearing butyl and phenyl groups. The cytotoxic activity of complexes **2** and **3** is higher than for some triorganotin (IV) complexes, which nowadays are reported to be the derivatives with the highest activity [58]. It can also be observed that the presence of organic groups attached to the tin (IV) ion is not compulsory for displaying cytotoxic activity since complex **4** also presents high activity in the four cancer cell lines. In general, the activity of the complexes increases as the lipophilycity of the organic substituents bound to the Sn (IV) centre increases. Complex **3** showed the highest efficacy in inhibiting proliferation of all the cancer cell lines studied (Figure 5(b), S38), with IC_50_ values for HeLa and MDA-MB231 lines lower than 1 *µ*M (highly cytotoxic), so this compound was selected for further experiments.

The microscopic images 20X (S39) illustrate the cytotoxic as well as cytostatic antiproliferative effect of complex **3** on MDA-MB-231 cell in a concentration-dependent manner. Thus, based on these results, this triple negative breast cancer cell line was selected for our further study.

### 3.6. Mediated Inhibition in Cell Cycle Progression by Inducing G2/M Arrest

**Figure 6:**
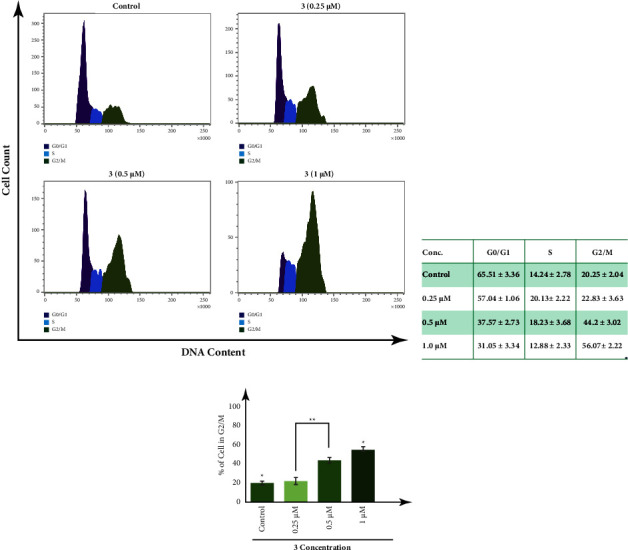
FACS analysis (a) and table (b) showing concentration-dependent increase in MDA-MB-231 G2/M cell population, in response to treatment with different concentrations of complex **3**. (c) Histogram representing dose-dependent increase in the proportion of MDA-MB-231 cells in the G2/M phase along with decrease in the G1 phase in response to complex **3**. All the experiments have been repeated thrice and the results were expressed as the mean ± SEM. The level of significance was set at ^*∗∗∗*^*P* < 0.001; ^*∗∗*^*P* ≤ 0.01 − 0.001; and ^*∗*^*P* ≤ 0.01 − 0.05 in respect to the control.

We made flow cytometric analysis to further understand how complex **3** affects the cell cycle distribution profile of MDA-MB-231 cells, as well as the antiproliferative mechanism. Interestingly, flow cytometric data clearly show a significant level of increase in the G2/M phase cell proportion in response to different concentrations of complex **3**, accompanied by a significant reduction in G1 phase cell population, and show a dose-dependent increase in induction of G2/M arrest, and at a concentration 1 *µ*M show G2/M arrest of 56.07% of cells population (Figure 6).

### 3.7. Cell Death

**Figure 7:**
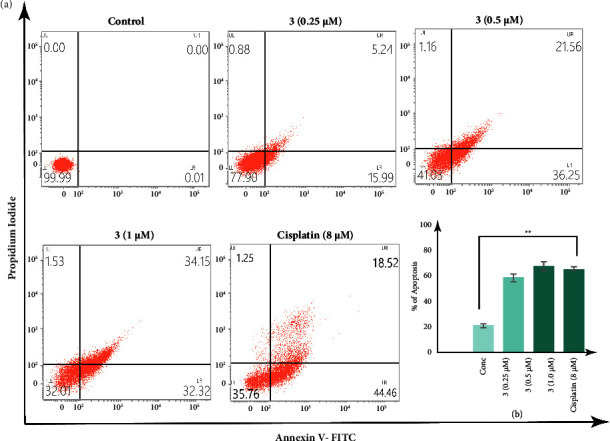
FACS analysis (a) showing concentration-dependent increase in annexin V-FITC/PI-positive population of MDA-MB-231 cells, in response to treatment with complex **3** and cisplatin. (b) Histograms representing dose-dependent increase in the apoptotic cell population percentage in response to complex **3** and cisplatin. All the experiments have been repeated thrice and the results were expressed as the mean ± SEM. The level of significance was set at ^*∗∗∗*^*P* < 0.001; ^*∗∗*^*P* ≤ 0.01 − 0.001; and ^*∗*^*P* ≤ 0.01 − 0.05 in respect to the control.

To better understand whether complex **3** mediated inhibition of MDA-MB-231 cells proliferation is related to apoptotic or necroptotic cell death, annexin V-FITC/PI flow cytometric assay was used to settle the mode of cell death, using cisplatin (8 *µ*M) as the positive control. The data show a higher number of apoptotic cell population with the increase of complex **3** concentration. As shown in Figure 7, 0.25 *µ*M, 0.5 *µ*M, and 1 *µ*M treatment with complex **3** for 24 h increased the annexin-V-FITC^+^/PI^−^ (early apoptotic) and annexin V-FITC^+^/PI^+^ (late apoptotic) cell population to 21.23%, 57.81%, and 66.47%, respectively, from 0.01% in the control setup. At a concentration of compound **3** of 0.25 *µ*M shows an early apoptotic cell population of 15.99% and late apoptotic cell population of 5.24%, at a concentration of 0.5 *µ*M shows an early apoptotic cell population of 36.25% and late apoptotic cell population of 21.56%, and at a concentration of 1 *µ*M shows an early apoptotic cell population of 32.32% and late apoptotic cell population of 34.15%, while cisplatin at a concentration of 8 *µ*M shows an early apoptotic cell population of 44.46% and late apoptotic cell population of 18.52%. No significant cell population has been found in the necrotic cell population zone, which confirms that complex **3** mediates its action by targeting the apoptotic cell death pathway and even at a much lover concentration of 1 *µ*M, complex **3** exerts better apoptotic activity that cisplatin.

### 3.8. Intracellular Reactive Oxygen Species Accumulation

**Figure 8:**
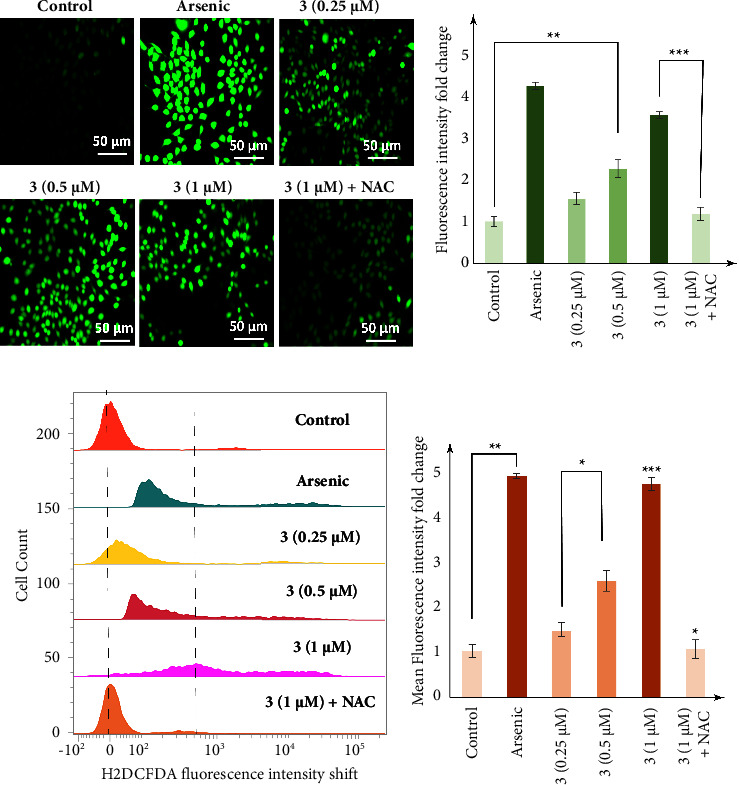
Concentration-dependent increase in intracellular ROS accumulation of MDA-MB-231 cells, in response to treatment with complex **3**. Post treatment with **3** (0.25 *µ*M, 0.5 *µ*M, and 1 *µ*M) for 24 h, cells were stained with H_2_DCFDA. (a) showing fluorescence microscopic analysis, (a) and (b) showing fold increase in fluorescence intensity, and (c) showing FACS analysis, where *X* axis denotes cell count where each experimental condition has normalized cell population of 200. (c) and (d) showing fold change in mean fluorescence intensity. The level of significance was set at ^*∗∗∗*^*P* < 0.001; ^*∗∗*^*P* ≤ 0.01 − 0.001; and ^*∗*^*P* ≤ 0.01 − 0.05 in respect to the control.

To find out whether apoptotic cell death mediated by complex **3** was promoted by accumulation of intracellular reactive oxygen species (ROS), we used H_2_DCFDA fluorescence assay, and ROS generation was measured using fluorescence microscopic and flow-cytometric analysis. Exposure to arsenic induces the generation of ROS, so it has been used as a positive control. Fluorescence microscopy images of cells treated with **3** show a dose-dependent increase in the level of green colour fluorescence intensity as compared to control untreated cells, showing intracellular ROS accumulation even at a low concentration of 0.5 *µ*M. Furthermore, at a concentration of 1 *µ*M, complex **3** shows 3.57 ± 0.08-fold increase in fluorescence intensity in comparison to control, which was close to arsenic-induced fold increase of green fluorescence intensity 4.28 ± 0.09 (Figures 8(a) and 8(b)). Nonetheless, MDA-MB-231 cells pretreated with 10 mM N-acetylcysteine (NAC), a potent and widely accepted inhibitor of ROS, reduce the generation of ROS induced by complex **3** in comparison to treatment with complex **3** alone at a concentration of 1 *µ*M (Figure 8).

In addition, flow cytometric data also show a dose-dependent increase in the level of mean fluorescence intensity (MFI) in cells treated with complex **3**. As shown in Figure 8, treatment with arsenic (1 *µ*M) and complex **3** at a concentration of 0.25 *µ*M, 0.5 *µ*M, and 1 *µ*M for 24 h increased the mean fluorescence intensity (MFI) to 4.91 ± 0.05, 1.47 ± 0.17, 2.58 ± 0.23, and 4.73 ± 0.14, respectively, in comparison with the control setup. Further treatment with complex **3** at 1 *µ*M in NAC pretreated cells shows a decrease in the MFI value. Altogether, the above observed results clearly indicate that intracellular ROS accumulation participates in apoptotic cell death mediated by complex **3**.

### 3.9. Disruption of Mitochondrial Membrane Potential

**Figure 9:**
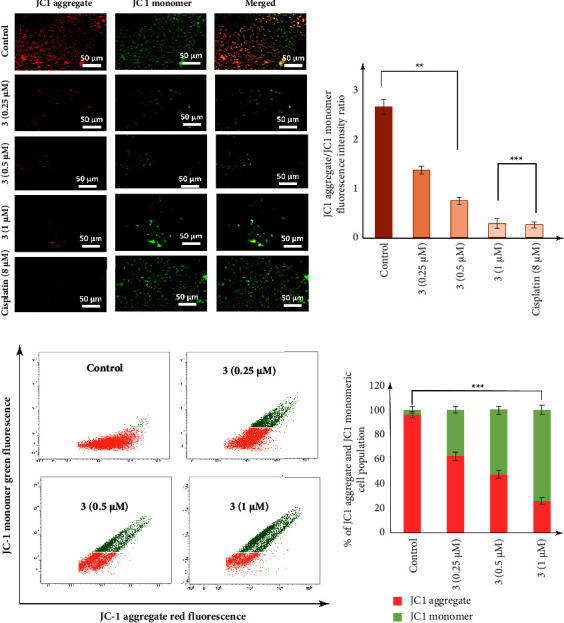
Dose-dependent disruption in mitochondrial membrane permeability (MMP) in MDA-MB-231 cells treated with complex **3**. Post treatment with **3** (0.25 *µ*M, 0.5 *µ*M, and 1 *µ*M) for 24 h, cells were stained with JC1. (a) fluorescence microscopic analysis, and (a) and (b) fold increase in JC1 aggregate/JC1 monomer fluorescence intensity ratio. (c) FACS analysis and (d) percentage of JC1 aggregate and JC1 monomer cell population.

Since mitochondrial membrane permeability (MMP) transition plays a central coordinating role in apoptosis induction, we further analyzed whether mitochondrial membrane potential was disrupted in MDA-MB-231 cells after treatment with complex **3**. MMP was measured by JC1 staying using both fluorescence microscopy and flow-cytometric methods. The cyanine dye JC1 exhibits potential-dependent accumulation in mitochondria, indicated by a shift in the fluorescence emission pattern from red (JC1 aggregate) to green (JC1 monomer) as the mitochondrial membrane is damaged and loses its potential. The results (Figure 9) show a dose-dependent increase in green fluorescence intensity or decrease in the JC1 aggregate/JC1 monomer fluorescence intensity ratio in response to treatment with complex **3** after 24 hours. Compound **3** at a concentration of 0.25 *µ*M shows a JC1 aggregate/JC1 monomer fluorescence intensity ratio of 1.36 ± 0.78, which decreases to the ratio of 0.27 ± 0.13 at a concentration of 1 *µ*M of complex **3**, in comparison to control setup after 24 h of treatment. Furthermore, at a concentration of 8 *µ*M, cisplatin shows a JC1 aggregate/JC1 monomer fluorescence intensity ratio of 0.25 ± 0.06, indicating complex **3** have more efficient mitochondrial depolarization activity than cisplatin (Figures 9(a) and 9(b)).

Further flow cytometric analysis also shows a dose-dependent increase in green fluorescence intensity upon exposure to **3**, with an increase in JC1 green fluorescence intensity in 37.47%, 52.22%, and 74.51% of the cellular population at a concentration of 0.25 *µ*M, 0.5 *µ*M, and 1 *µ*M, respectively, in comparison to 3.93% in control cell population after 24 h of treatment (Figures 9(c) and 9(d)). This observation hereby clearly indicates a disruption in the MMP of the MDA-MB-231 cells in response to treatment with complex **3**.

## 4. Conclusions

One new hybrid ligand derived from 2,3-butanedione, thiosemicarbazide, and isonicotinic acid hydrazide has been synthesized with high purity and yield. The possibility of protonation of the pyridine nitrogen affords the ligand in both its neutral and cationic form although no significant differences were observed in the reactivity of the two forms of the ligand. From the reaction with SnR_2_Cl_2_ (*R* = Me, Bu, and Ph) and tin (IV) iodide, four new coordination compounds were isolated. Reaction with SnR_3_Cl (*R* = Me, Bu, and Ph) failed in obtaining complexes containing the hybrid thiosemicarbazone/hydrazone ligand, and only complexes with the triazine formed by ligand reorganization were obtained, so further experiments should be planned to obtain the desired complexes. The organometallic derivatives **1–3** have a polymeric structure in the solid state that is lost in solution to afford monomeric species.

All the compounds exhibit higher antiproliferative activity than cisplatin against HeLa, MDA-MB-231, HepG2, and PC3 cancer cell lines. The most active compound, containing phenyl substituents, has been selected for further experiments on MDA-MB-231 cell line to determine its mode of action. The results clearly show that the complex can effectively induce apoptotic cell death by the accumulation of reactive oxygen species and disruption of mitochondrial membrane potential.

## Figures and Tables

**Table 1:** Comparative IC_50_ values of L^1^H_2_, [L^1^H_3_]Cl, **1**, **2**, **3**, and **4** (0–5 *µ*M) in cancer cell lines MDA-MB-231, HeLa, PC3, and HepG2 and normal lung fibroblast cell line WI-38.

Compounds	HeLa	MDA-MB231	HepG2	PC3	WI-38
**L** ^**1**^ **H**_**2**_	1.06 ± 0.21	2.42 ± 0.33	5.14 ± 0.52	4.86 ± 0.75	10.78 ± 0.96
**[L** ^**1**^ **H**_**3**_ **]Cl**	1.49 ± 0.46	2.48 ± 0.47	6.27 ± 0.98	3.49 ± 0.73	14.09 ± 0.48
**1**	2.22 ± 0.54	3.54 ± 0.34	4.97 ± 0.76	2.76 ± 0.52	15.63 ± 0.73
**2**	0.78 ± 0.27	1.038 ± 0.21	2.46 ± 0.42	2.58 ± 0.36	12.02 ± 0.89
**3**	0.43 ± 0.18	0.51 ± 0.13	1.59 ± 0.17	1.97 ± 0.24	15.69 ± 0.28
**4**	2.19 ± 0.36	1.78 ± 0.26	3.73 ± 0.43	4.29 ± 0.29	14.38 ± 0.28
**Cisplatin**	3.26 ± 0.23	8.25 ± 1.64	11.49 ± 2.06	5.36 ± 1.24	20.56 ± 1.52

## Data Availability

The data used to support the findings of this study are available in the supplementary material of this article.

## Supplementary Materials

Supplementary Materials Mass spectra, crystallographic data, IR, ^1^H, ^13^C, and ^119^Sn NMR spectra, percentage of cell viability, and microscopic images (20X) are included in the supplementary material.
