# Supplementary material for: New Tin (IV) and Organotin (IV) Complexes with a Hybrid Thiosemicarbazone/Hydrazone Ligand: Synthesis, Crystal Structure, and Antiproliferative Activity
Source: Bioinorg Chem Appl. 2024 Apr 3;2024:1018375. doi: 10.1155/2024/1018375 (PMC11006503; doi:10.1155/2024/1018375)
Supplement: Supplementary Materials — Mass spectra, crystallographic data, IR, 1H, 13C, and 119Sn NMR spectra, percentage of cell viability, and microscopic images (20X) are included in the supplementary material. [file 1018375.f1.docx]

**S1.** Atom labelling for NMR assignment


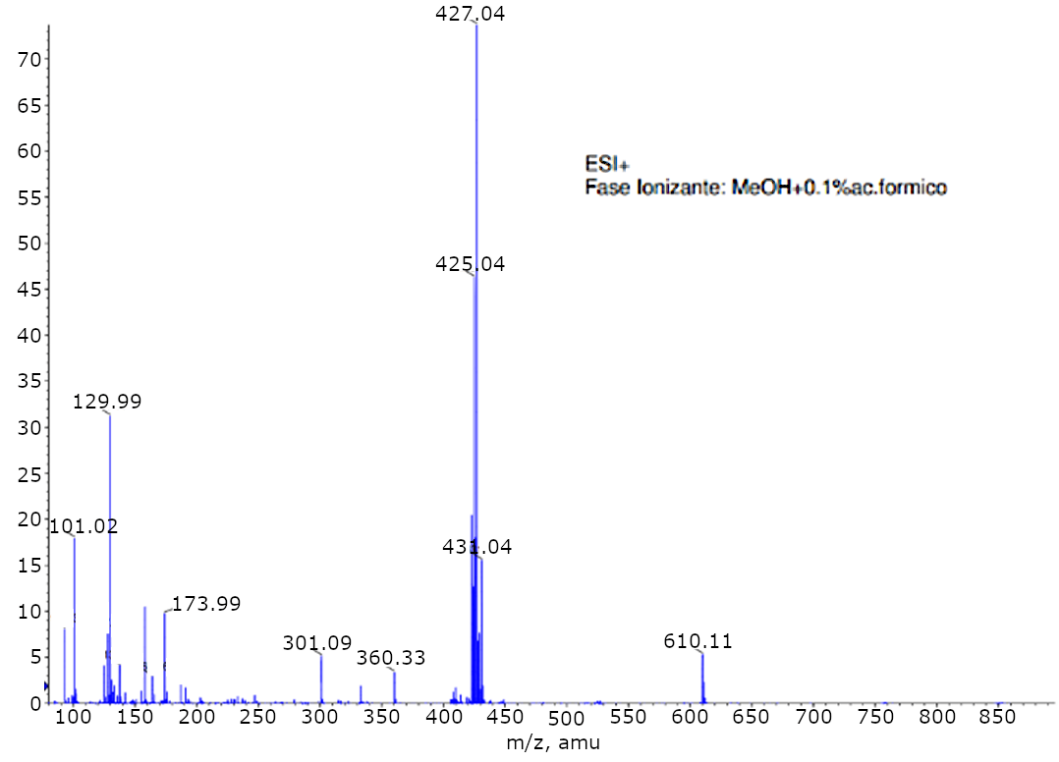

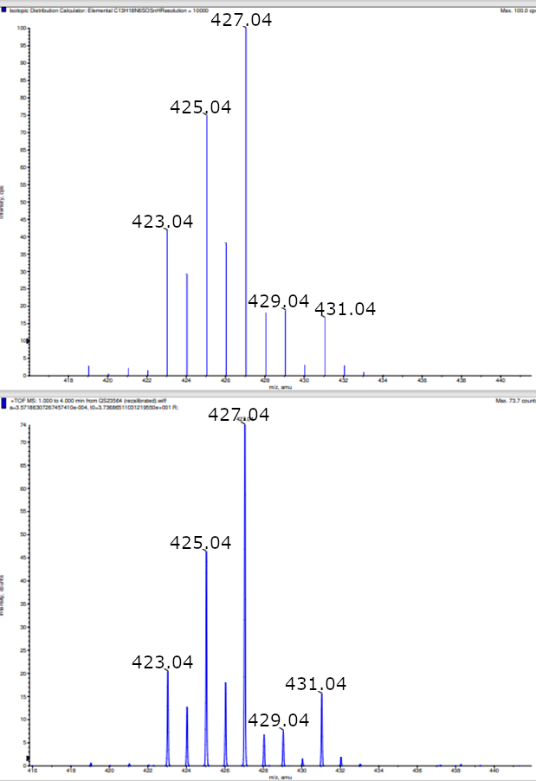


**S2.** Mass spectrum of complex **1** with theoretical (up) and experimental (down) isotopic pattern of the peak at 427.04 amu.


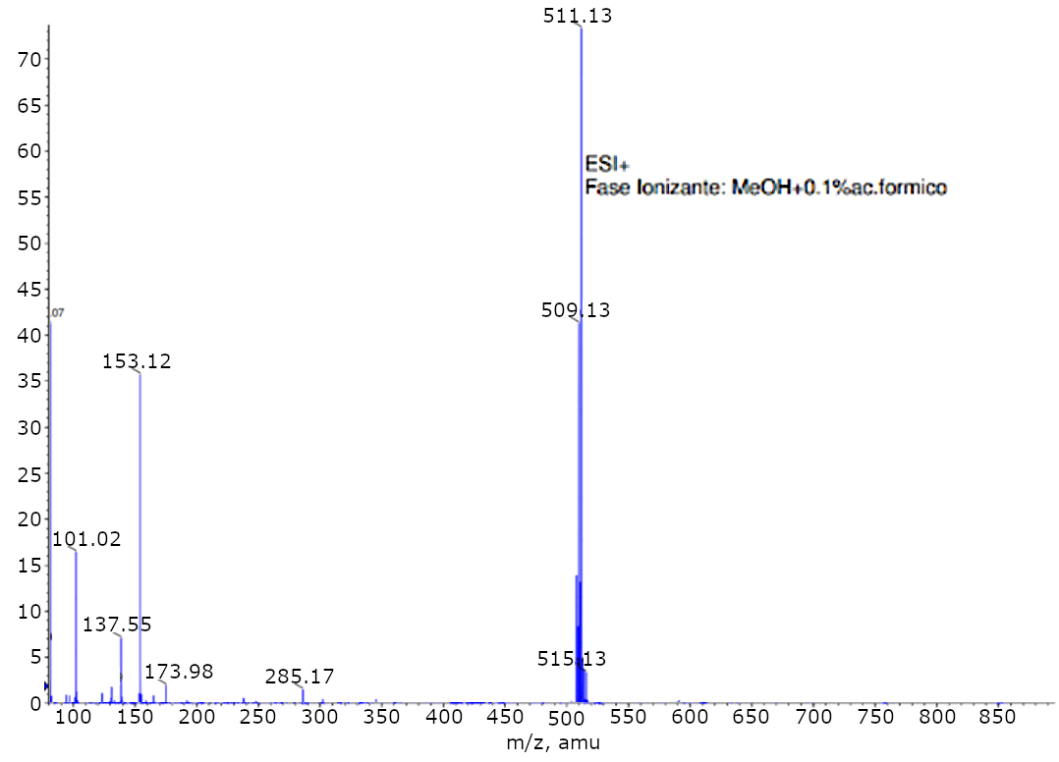

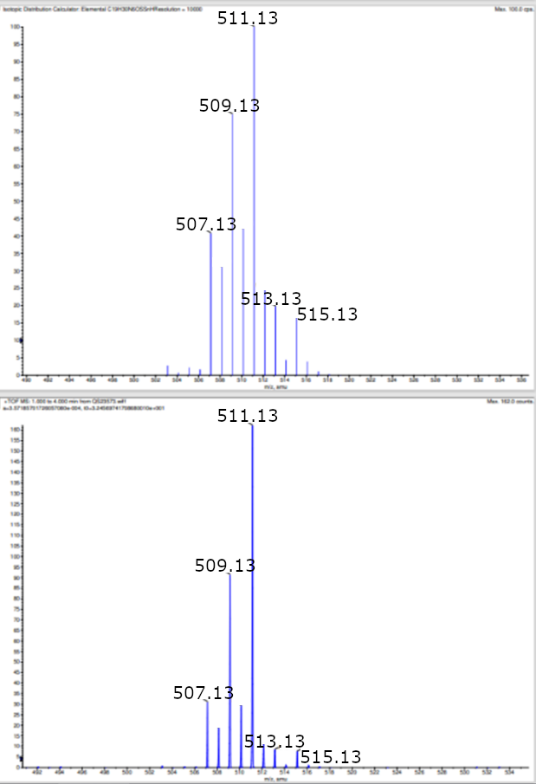


**S3.** Mass spectrum of complex **2** with theoretical (up) and experimental (down) isotopic pattern of the peak at 511.13 amu.


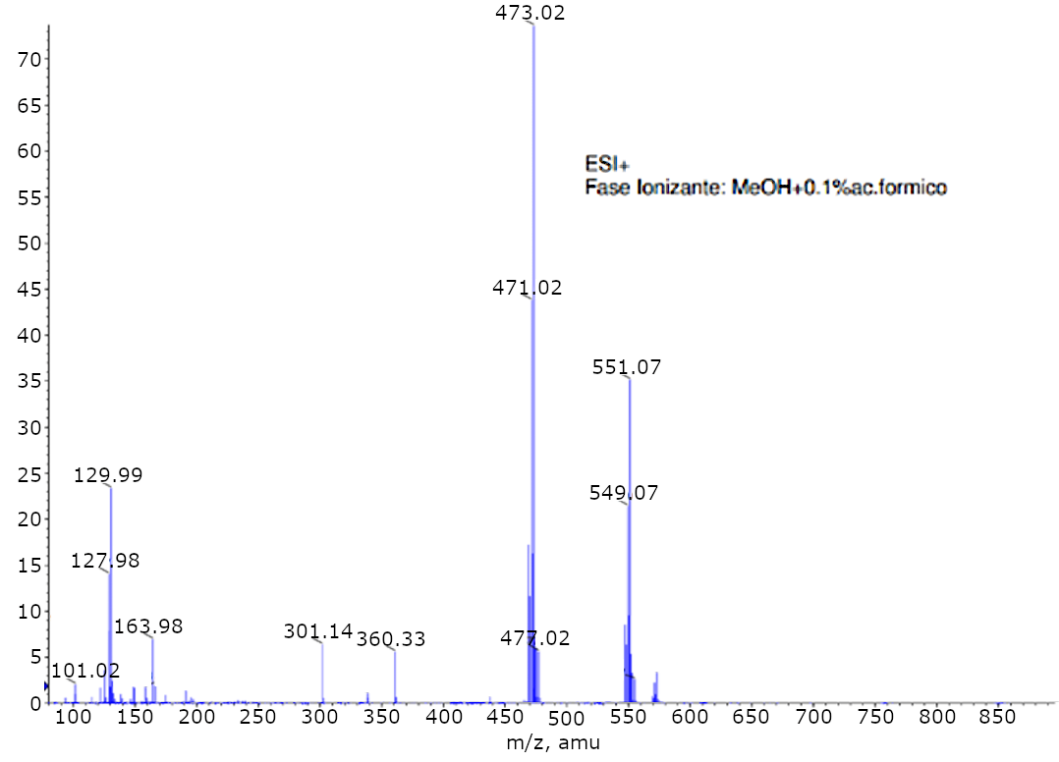

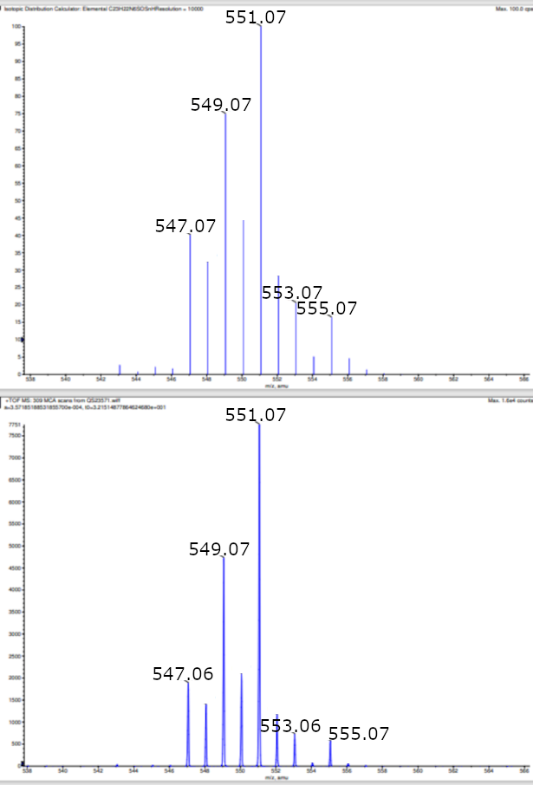


**S4.** Mass spectrum of complex **3** with theoretical (up) and experimental (down) isotopic pattern of the peak at 551.07 amu.


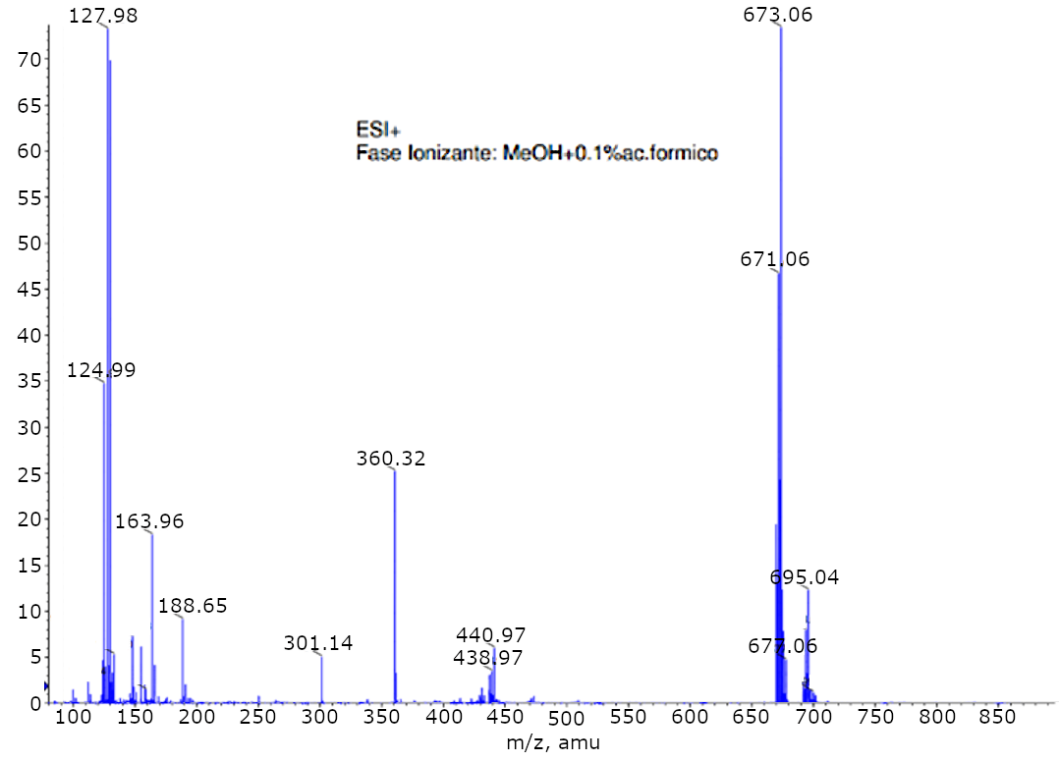

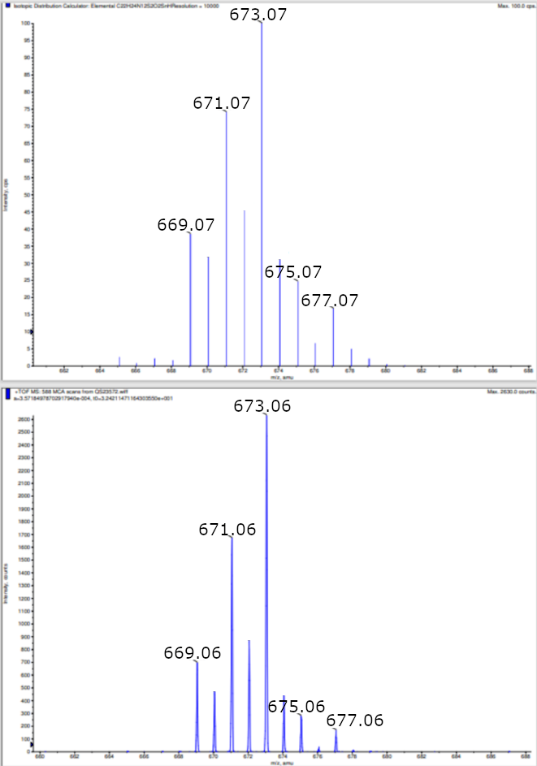


**S5.** Mass spectrum of complex **4** with theoretical (up) and experimental (down) isotopic pattern of the peak at 673.06 amu.

**S6.** Crystallographic and refinement data of complexes **2** needle, **2** plate and **4**.

|  | **2 needle** | **2 plate** | **4** |
| --- | --- | --- | --- |
| Formula | SnC_19_H_30_N_6_OS | Sn_2_C_42_H_69_N_12_O_4_S_4_ | SnC_24_H_30_N_12_O_3_S_2_ |
| M | 509.24 | 1171.71 | 717.41 |
| Temperature/K | 250(2) | 250(2) | 200(2) |
| Crystal system | Trigonal | Triclinic | Monoclinic |
| Space group | R -3 :H | P -1c | P 21/c |
| a/Å | 37.162(4) | 9.0995(3) | 11.0422(7) |
| b/Å | 37.162(4) | 16.0924(6) | 9.2569(6) |
| c/Å | 8.9904(10) | 19.3087(5)) | 30.3097(15) |
| α/° | 90 | 76.6750(10) | 90 |
| β/° | 90 | 77.7100(10) | 99.262(2) |
| γ/° | 120 | 87.7570(10) | 90 |
| U/ Å^3^ | 10752(3) | 2688.11(14) | 3057.8(3) |
| Z | 18 | 2 | 4 |
| D_c_/Mgm^-3^ | 1.416 | 1.448 | 1.558 |
| Absorption coefficient mm^-1^ | 1.177 | 1.134 | 1.021 |
| F(000) | 4680 | 1202 | 1456 |
| Goodness of fit on F^2^ | 1.214 | 1.473 | 1.063 |
| Reflections collected | 15907 | 162764 | 48773 |
| Independent reflections | 4227 [R(int) = 0.1916] | 11095[R(int) = 0.0574] | 6233 [R(int) = 0.1041] |
| Final R1and wR2[I>2σ(I)] | 0.0823, 0.13127 | 0.0512, 0.1724 | 0.0504, 0.1104 |
| R indices (all data) | R1=0.2483,  wR2 = 0.2795 | R1 = 0.0666,  wR2 = 0.1917 | R1 = 0.0940,  wR2 = 0.1478 |
| Residual electron density (min,max) (eÅ^-3^) | -1.298, 2.661 | -2.370, 2.443 | -0.785, 1.096 |

**S7.** Selected bond distances of **2** needle, **2** plate and **4**.

|  | **2 needle** | **2 plate** | **4** |
| --- | --- | --- | --- |
| Sn(1)-O(1) | 2.296(10) | 2.353(3) | 2.172(4) |
| Sn(1)-S(1) | 2.703(5) | 2.6684(14) | 2.538(14) |
| Sn(1)-N(3) | 2.408(13) | 2.368(4) | 2.365(4) |
| Sn(1)-N(4) | 2.315(12) | 2.306(4) | 2.287(4) |
| Sn(1)-C(12) | 2.165(14) | 2.142(5) | - |
| Sn(1)-C(16) | 2.162(15) | 2.125(8) | - |
| Sn(2)-N(6) #1 | 2.615(13) | - | - |
| Sn(1)-N(12) | - | 2.621(4) | - |
| Sn(1)-O(2) | - | - | 2.176(4) |
| Sn(1)-S(2) | - | - | 2.5281(16) |
| Sn(1)-N(9) | - | - | 2.360(4) |
| Sn(1)-N(10) | - | - | 2.294(4) |
| Sn(2)-O(2) | - | 2.289(3) | - |
| Sn(2)-O(3) | - | 2.530(5) | - |
| Sn(2)-S(2) | - | 2.7191(13) | - |
| Sn(2)-N(9) | - | 2.364(4) | - |
| Sn(2)-N(10) | - | 2.292(4) | - |
| Sn(2)-C(31) | - | 2.123(6) | - |
| Sn(2)-C(35) | - | 2.133(6) | - |

Symmetry transformations used to generate equivalent atoms: #1 -y+2/3, x-y+1/3, z+1/3

**S8.** Hydrogen bonding in complex **2** plate.


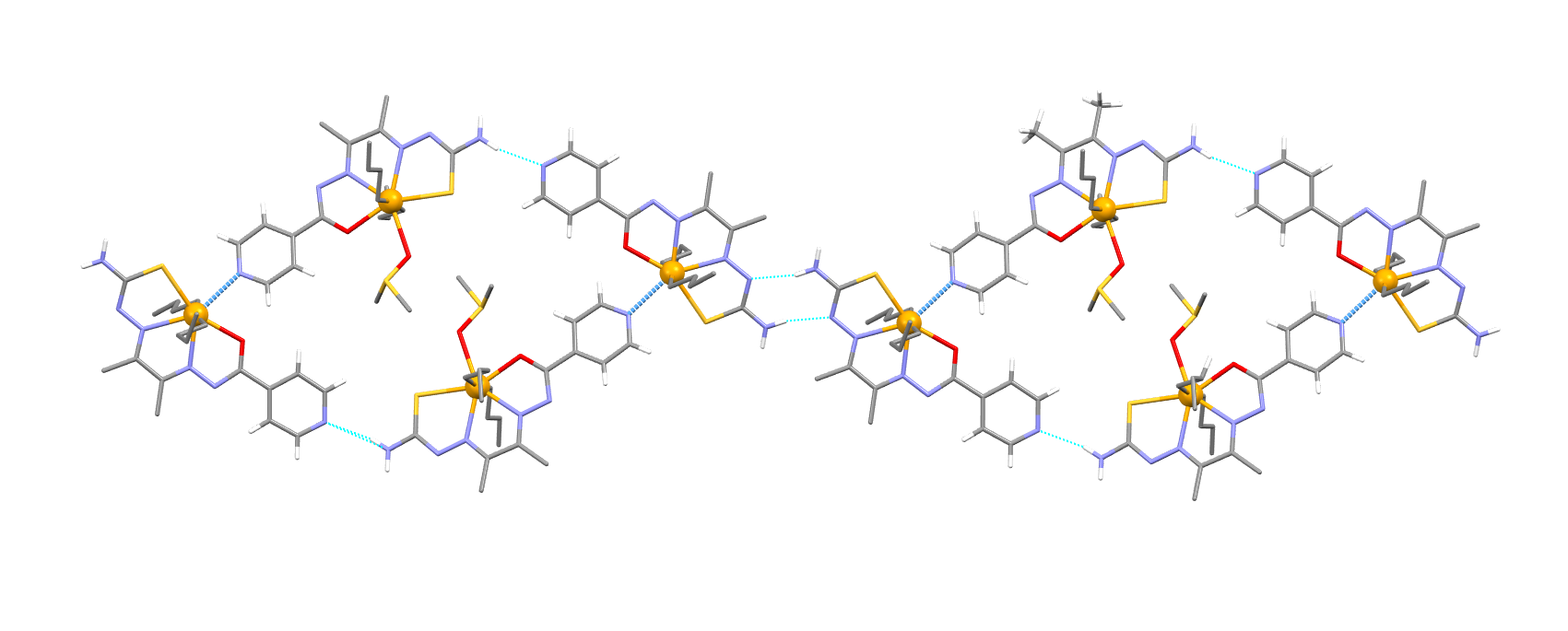


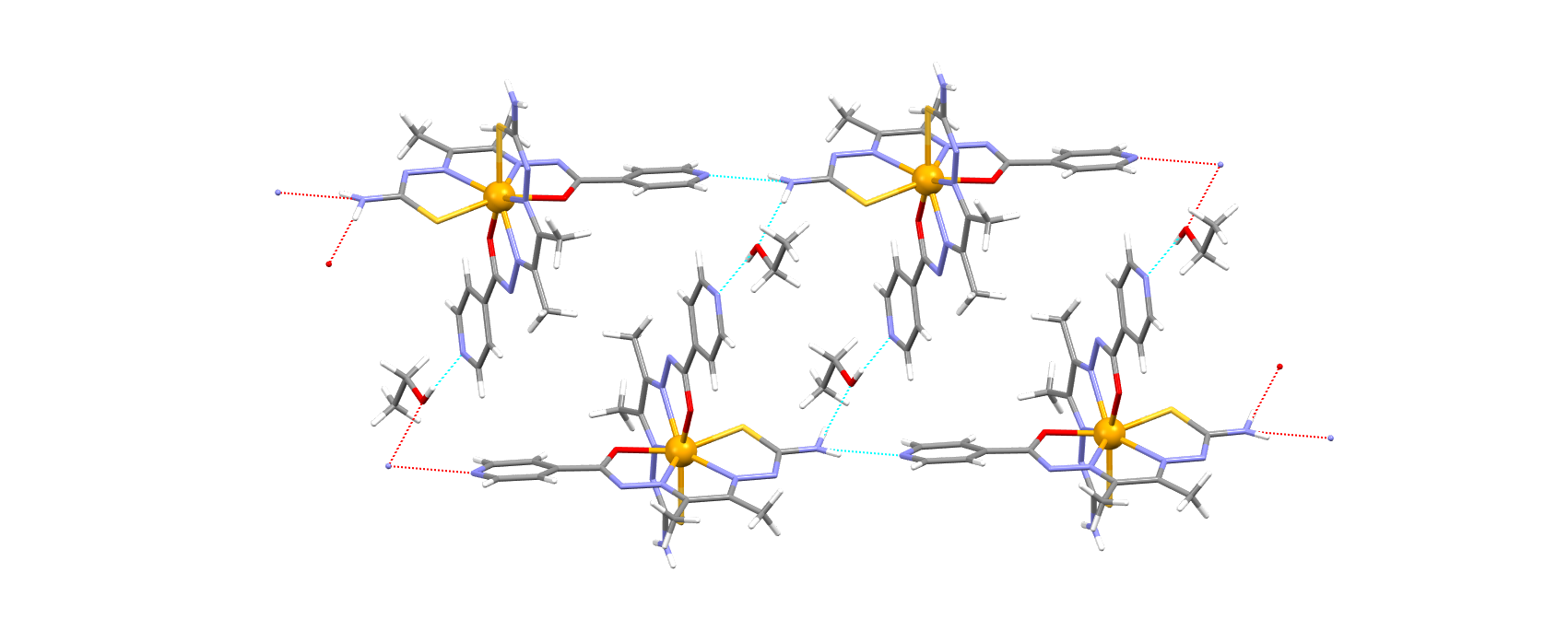


**S9.** Hydrogen bonding in complex [Sn(L^1^)_2_]·EtOH **4**

**S10.** Most important bands found in the IR spectra.

| **Compound** | **υ (N-H)** | **υ (C=O)** | **υ (C=N)** | **δ (H-N-H)** | **amide II**  **+**  **thioamide I** | **Thioamide IV** |
| --- | --- | --- | --- | --- | --- | --- |
| **[H_3_L^1^]Cl*** | 3442 (m)  3301 (m)  3152 (m) | 1671 (s) | 1634 (m) | as: 1606 (s)  s: 1385 (s) | 1542 (m) | 876 (m) |
| **H_2_L^1^** | 3369 (m)  3261 (m)  3169 (m) | 1670 (s)  1655 (s) | 1626 (m) | as: 1603 (s)  s: 1367 (m) | 1548 (m) | 851 (m) |
| **[SnMe_2_L^1^] (1)** | 3282.25 (m)  3168 (m) | 1671 (m) | 1561 (m) | as: 1608 (m)  s: 1368 (s) | 1523 (s) | 816 (w) |
| **[SnBu_2_L^1^] (2)** | 3395 (m) | 1632 (m) | 1572 (m) | as: 1604 (s)  s: 1368 (s) | 1523 (m) | 814 (w) |
| **[SnPh_2_L^1^] (3)** | 3444 (m)  3376 (m) | 1602 (m) | 1576 (m) | as: 1602 (m)  s: 1377 (s) | 1525 (m) | 816 (w) |
| **[Sn(L^1^)_2_]·EtOH (4)** | 3283 (m) | 1616 (m) | 15701 (m) | as: 1602 (m)  s: 1382 (m) | 1538 (m) | 817 (w) |

s: strong, m: medium, w: weak

* 2557 (s) υ (S-H)

**S11.** IR spectrum of L^1^H_2_ in KBr.

**S12.** IR spectrum of [L^1^H_3_]Cl in KBr.

**S13.** IR spectrum of complex **1** in KBr.

**S14.** IR spectrum of complex **2** in KBr.

**S15.** IR spectrum of complex **3** in KBr.

**S16.** IR spectrum of complex **4** in KBr.


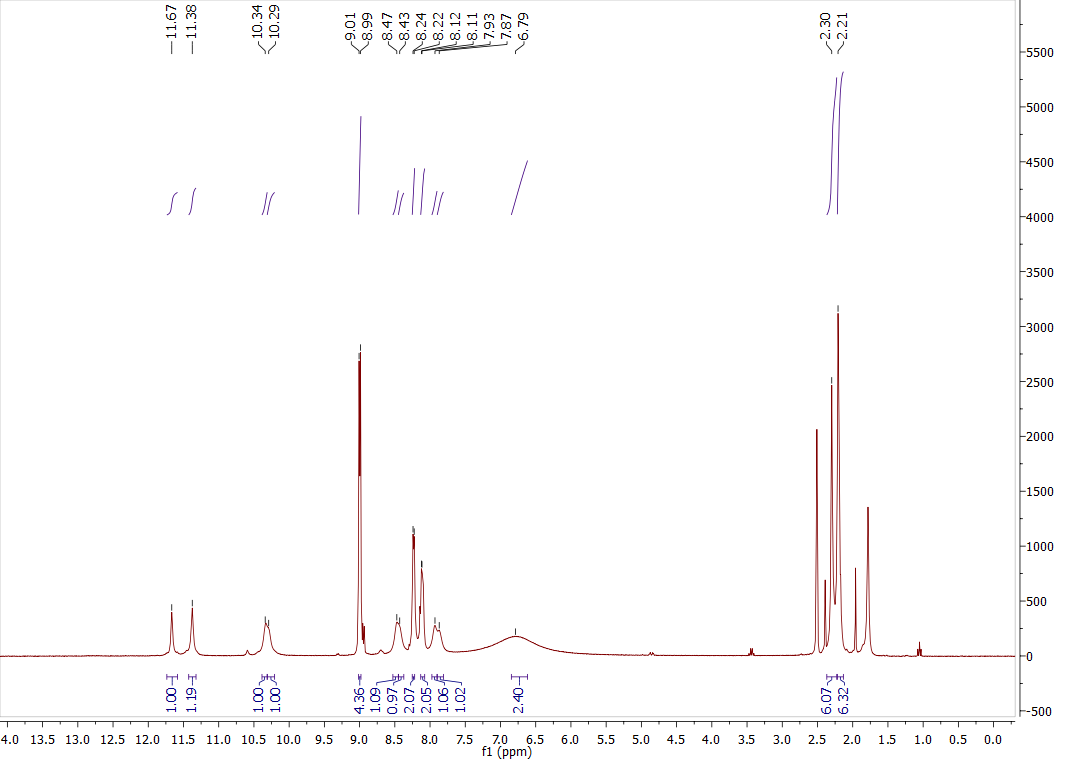


**S17.** ^1^H NMR spectrum of [L^1^H_3_]Cl in DMSO-d_6_.


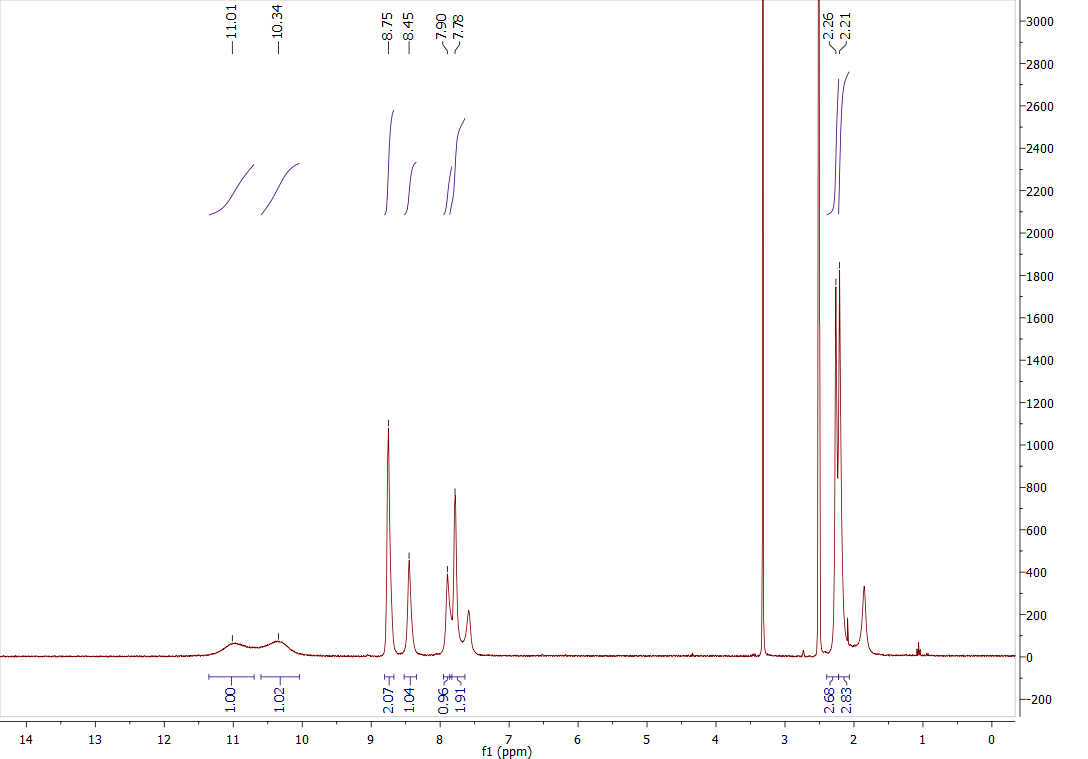


**S18.** ^1^H NMR spectrum of L^1^H_2_ in DMSO-d_6_.


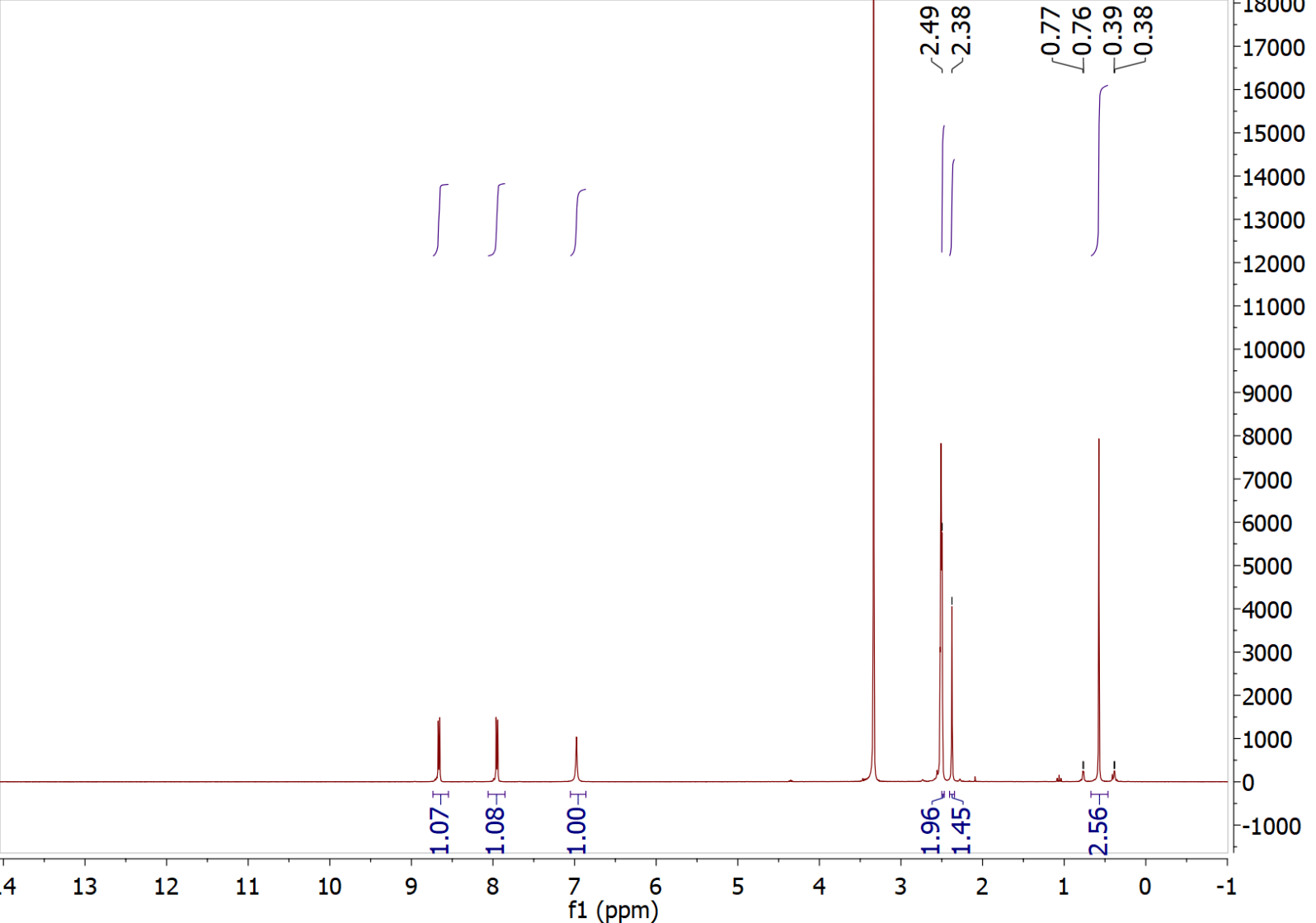


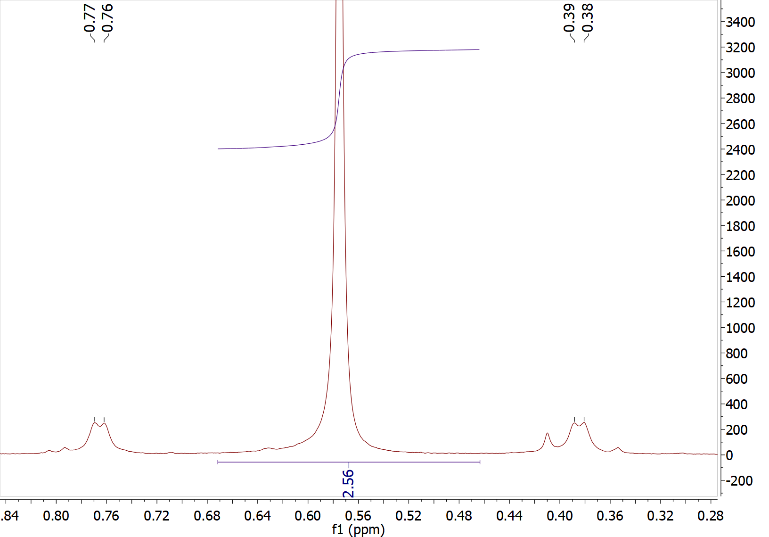

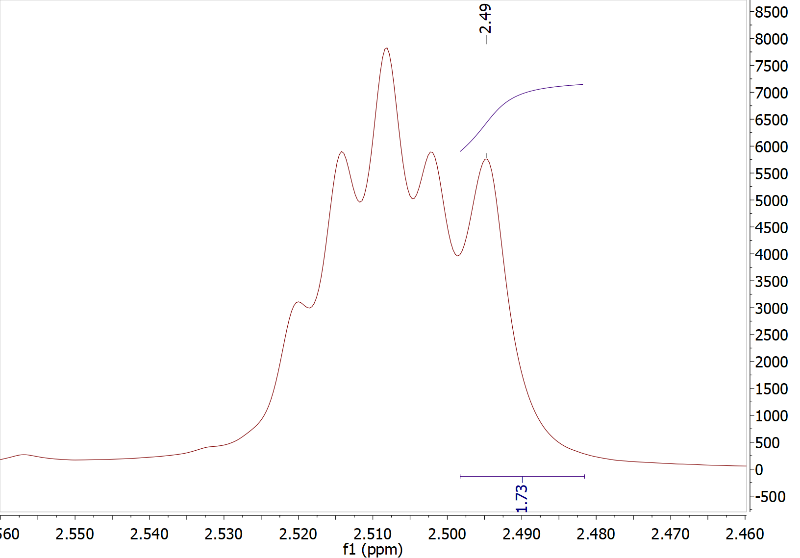


**S19.** ^1^H NMR spectrum of complex **1** in DMSO-d_6_ (up). View of the signals corresponding to CH_3_ groups of the ligand (down left) and to the CH_3_ groups bound to tin with the satellites corresponding to tin coupling (down right).


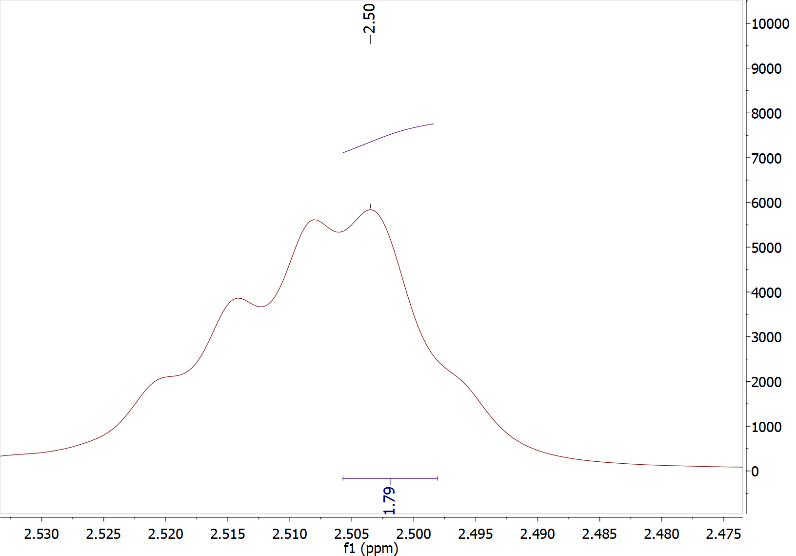

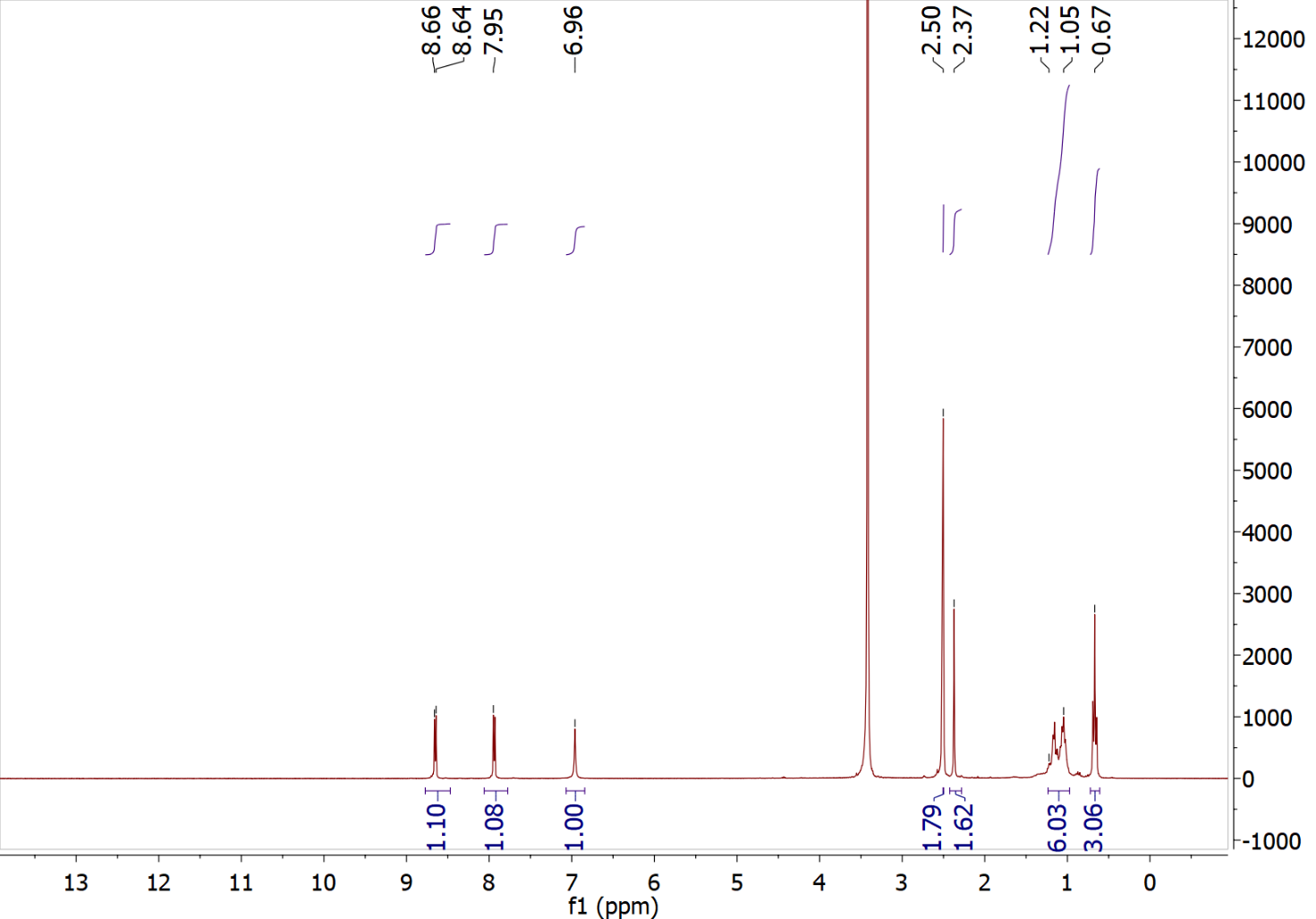
**S20.** ^1^H NMR spectrum of complex **2** in DMSO-d_6_ with view of the signal corresponding to one of the CH_3_ groups of the ligand (inset).


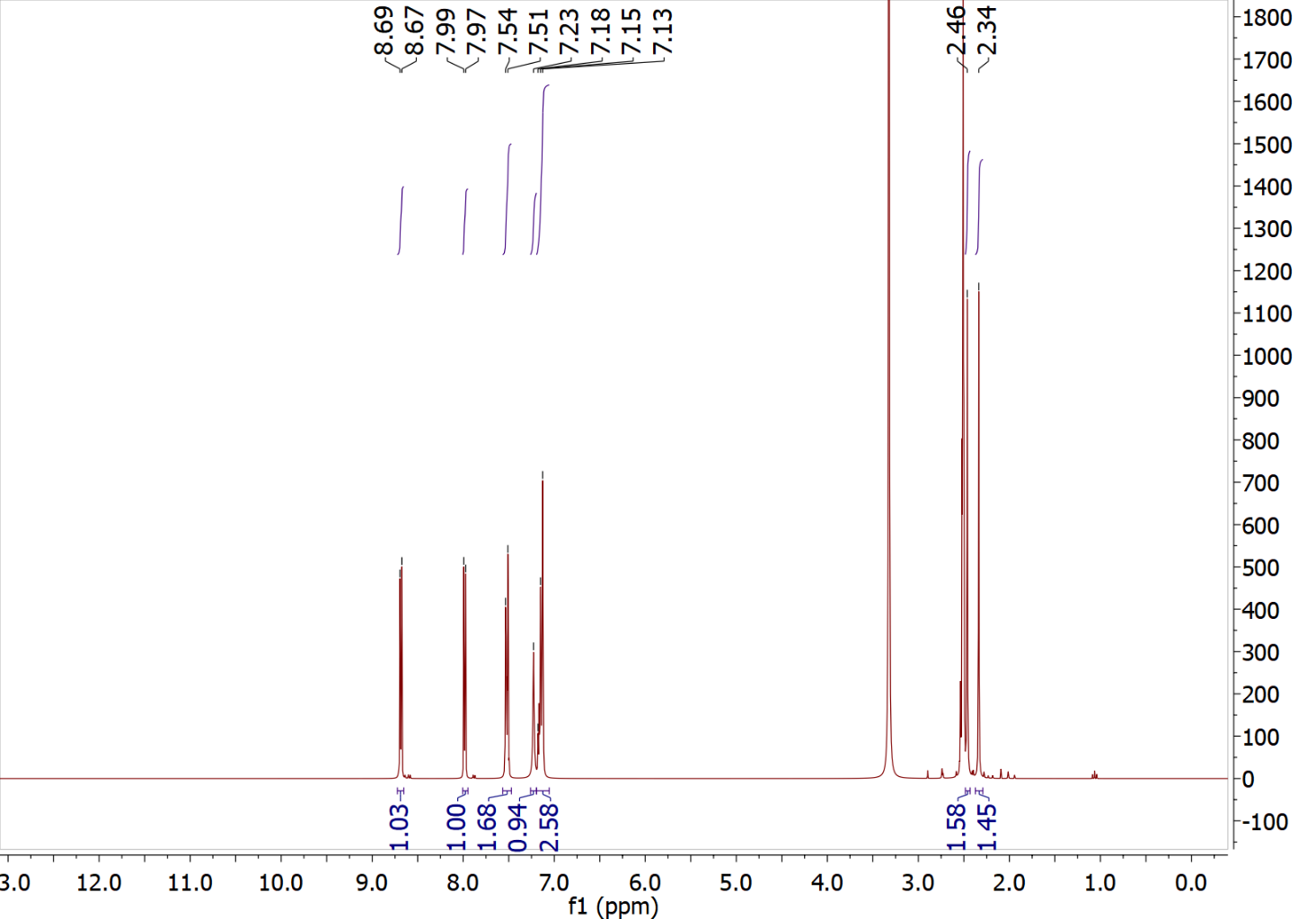


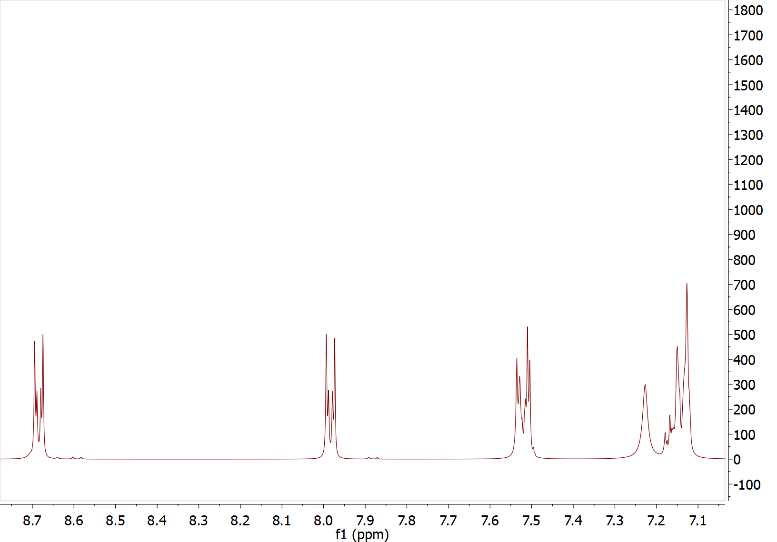


**S21.** ^1^H NMR spectrum of complex **3** in DMSO-d_6_ with view of the aromatic protons and the NH_2_ (inset).


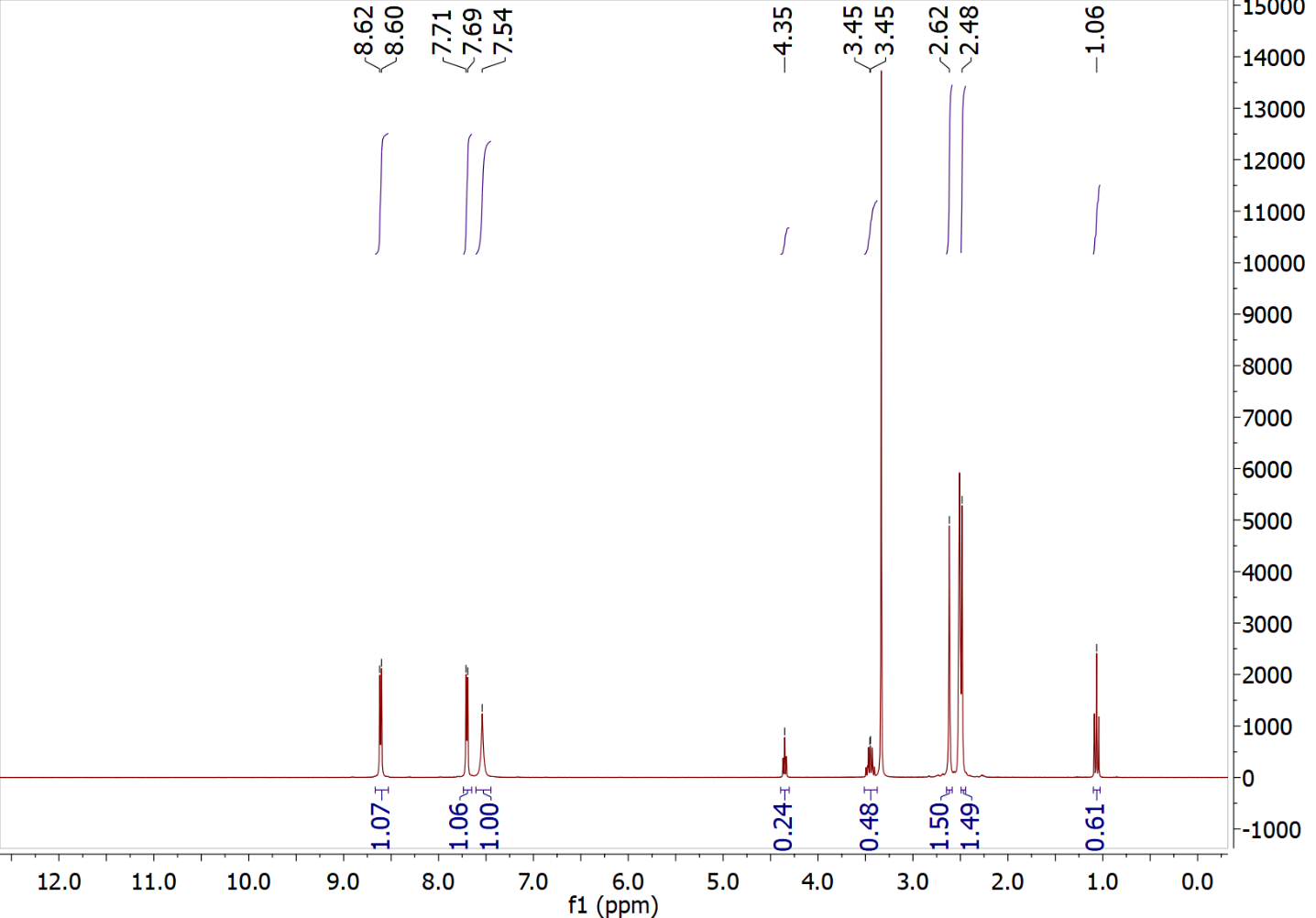


**S22.** ^1^H NMR spectrum of complex **4** in DMSO-d_6_.


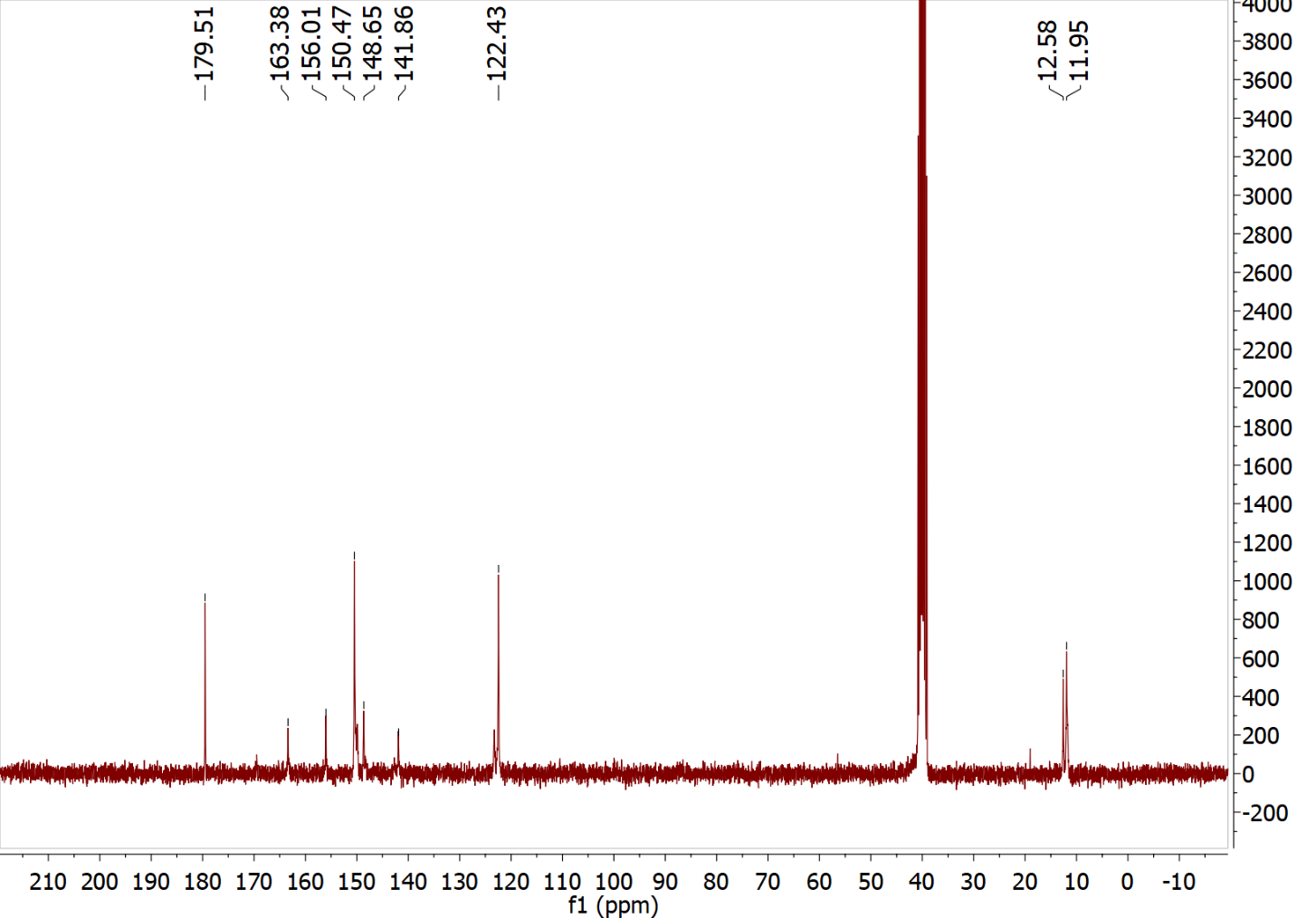


**S23.** ^13^C{^1^H} NMR spectrum of L^1^H_2_ in DMSO-d_6_.


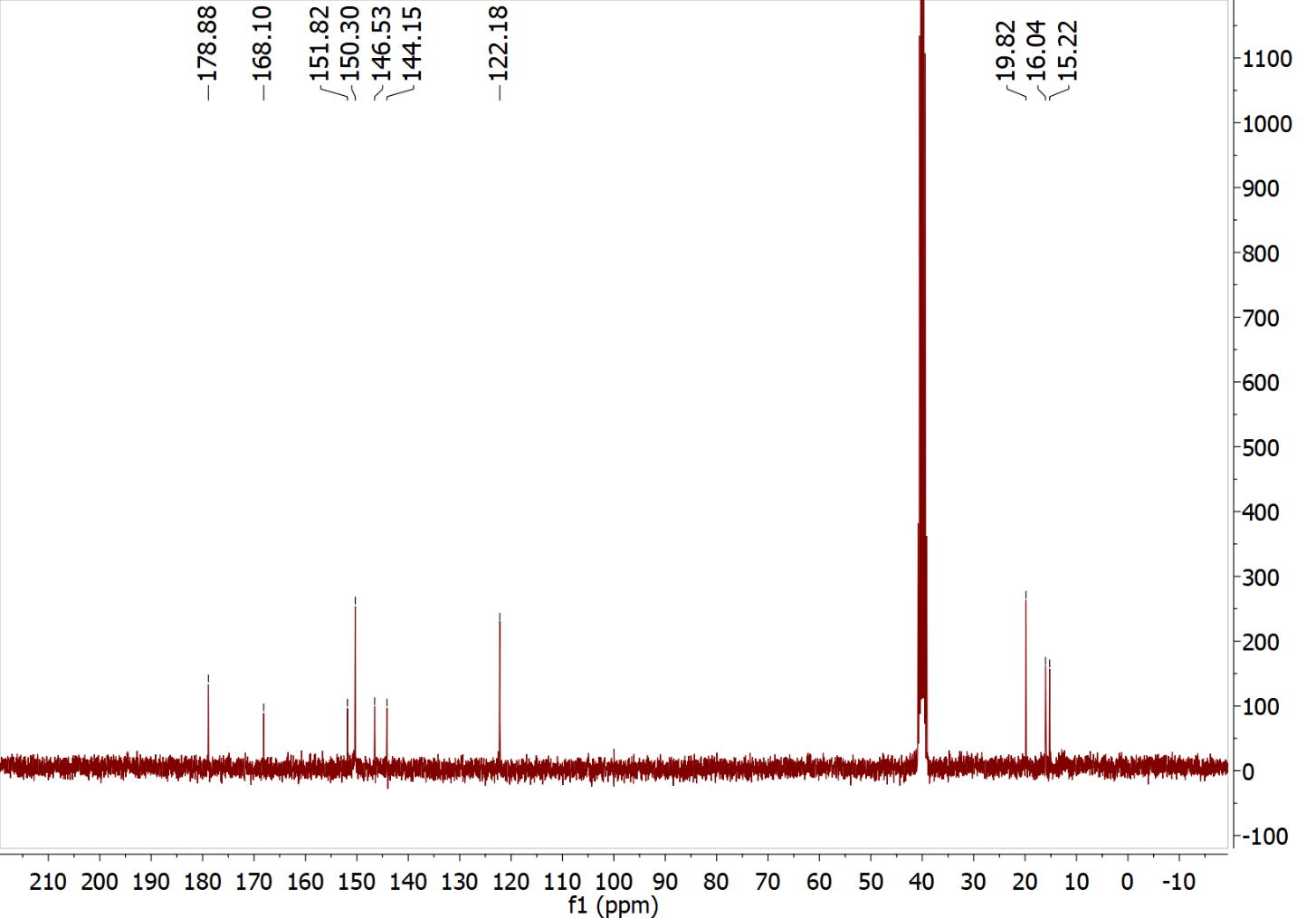


**S24.** ^13^C{^1^H} NMR spectrum of complex **1** in DMSO-d_6_.


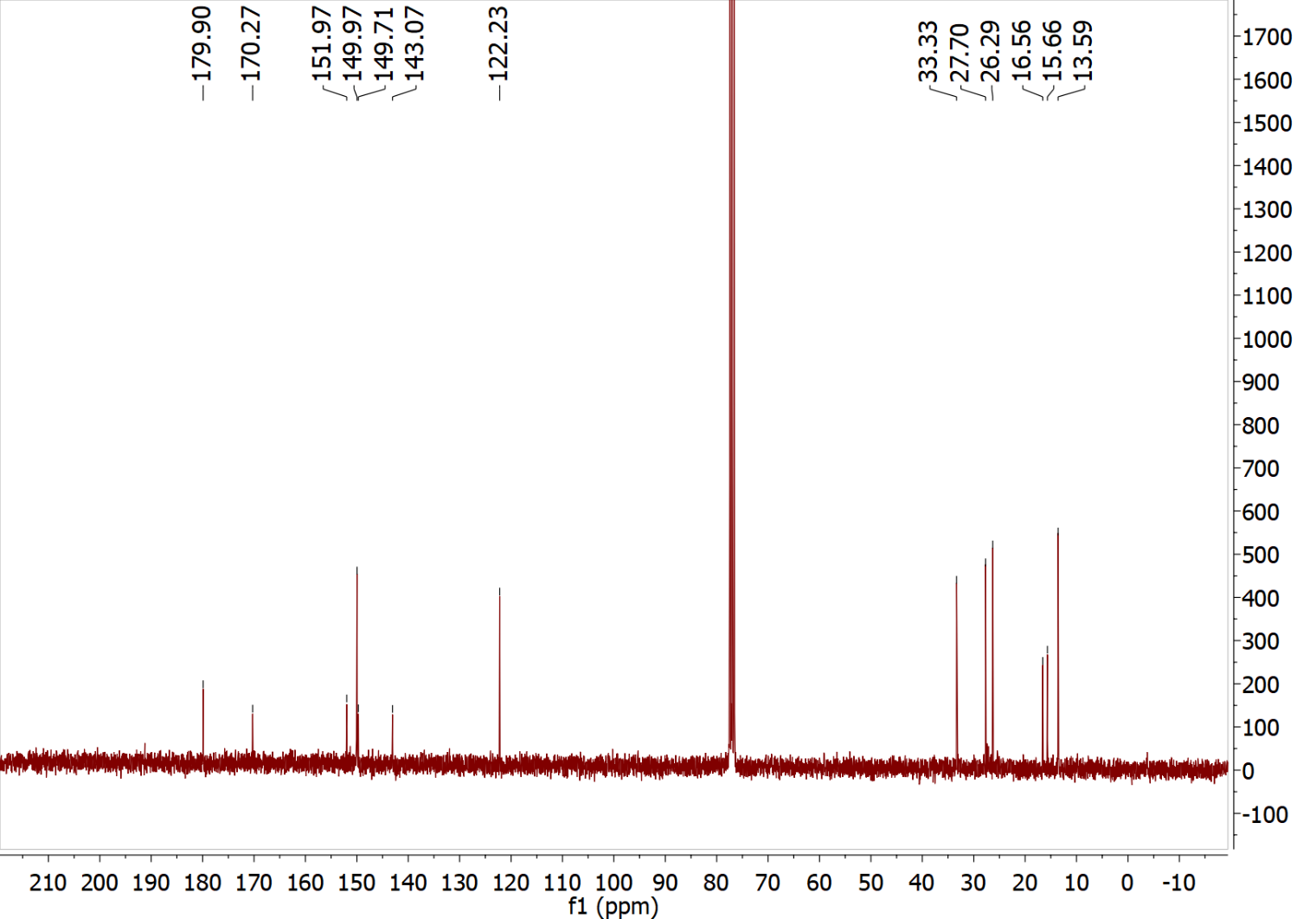


**S25.** ^13^C {^1^H} NMR spectrum of complex **2** in CDCl_3_.


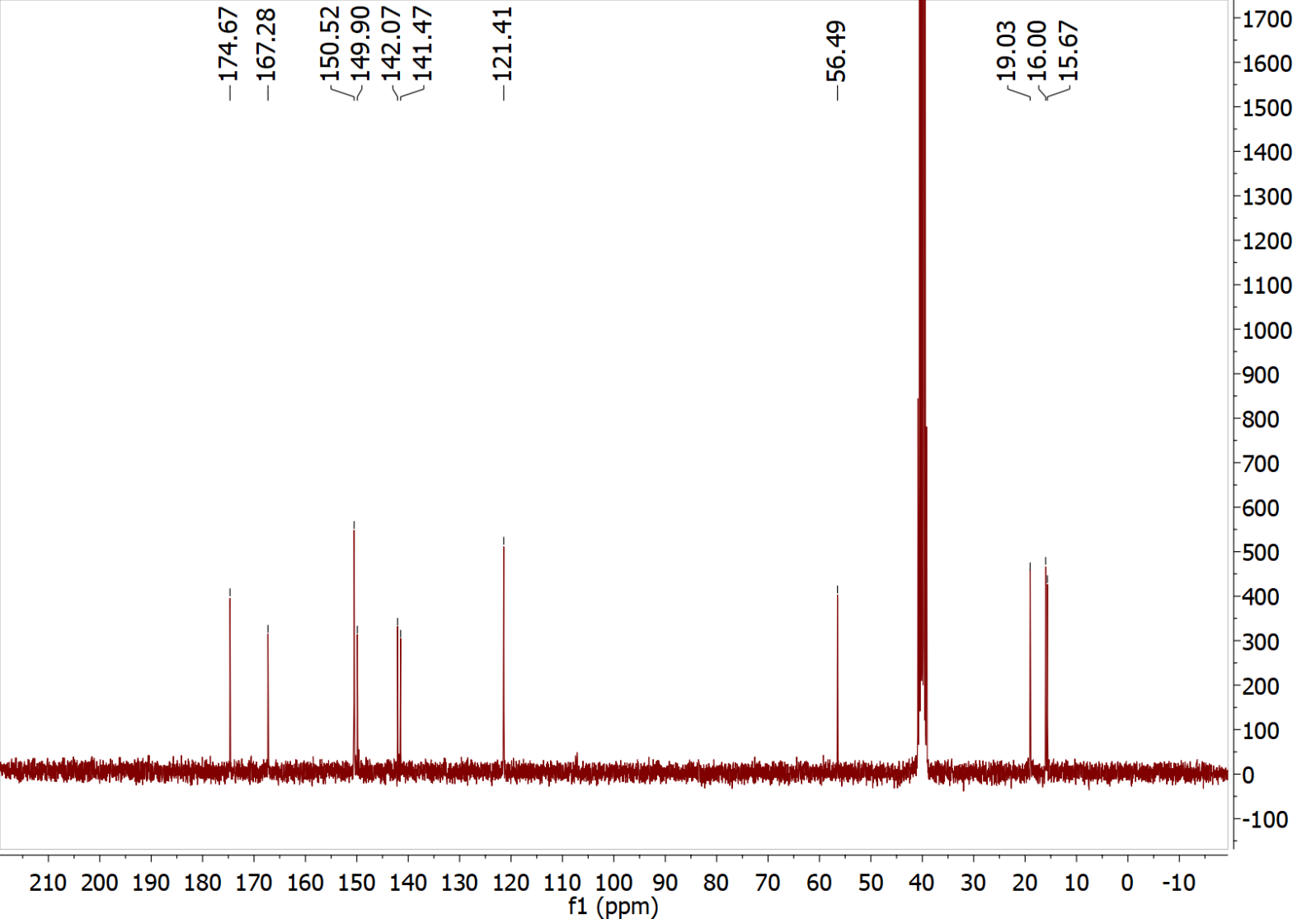


**26** ^13^C{^1^H} NMR spectrum of complex **4** in DMSO-d_6_.


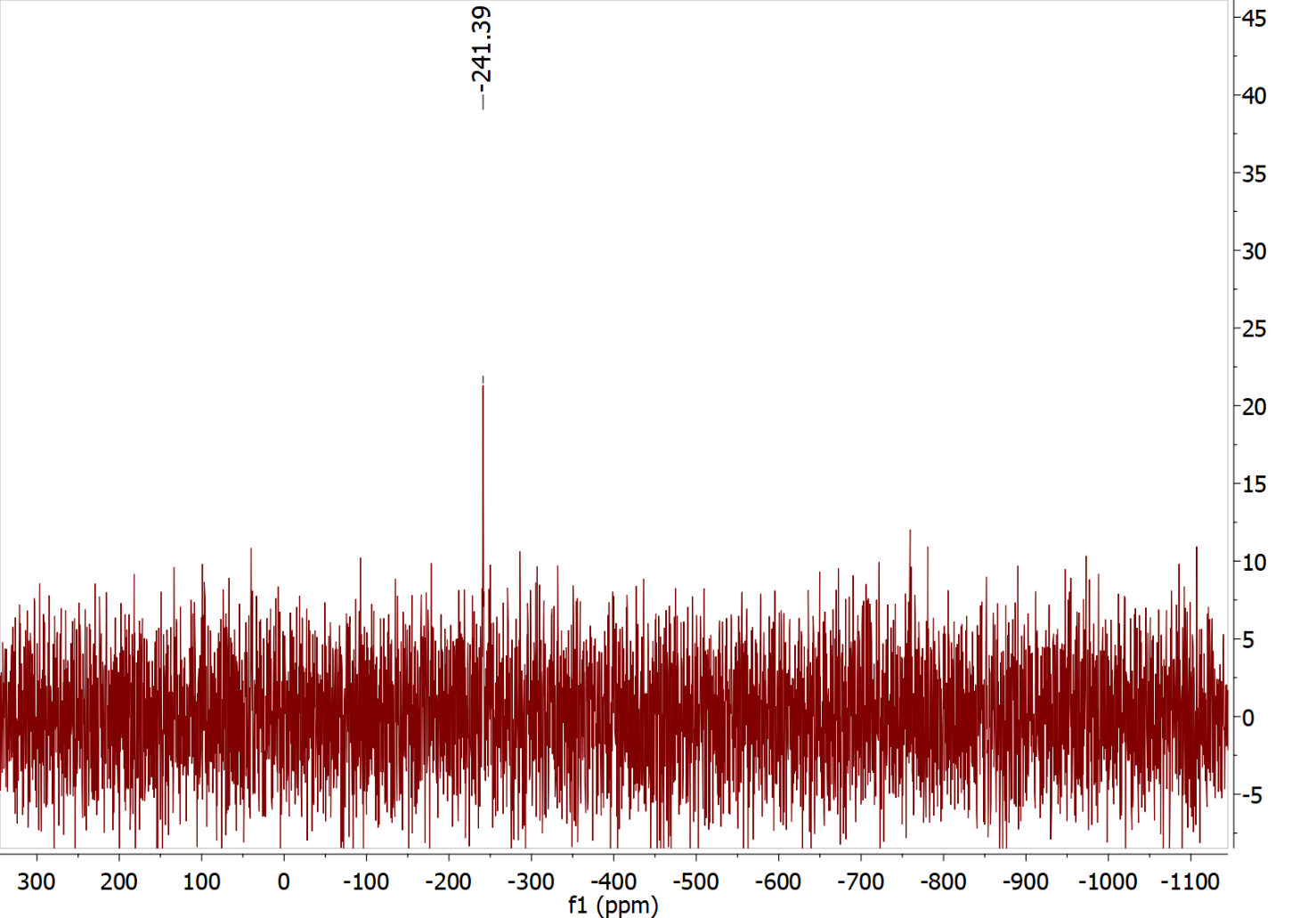
**S27.** ^119^Sn{^1^H} NMR spectrum of complex **1** in DMSO-d_6_.


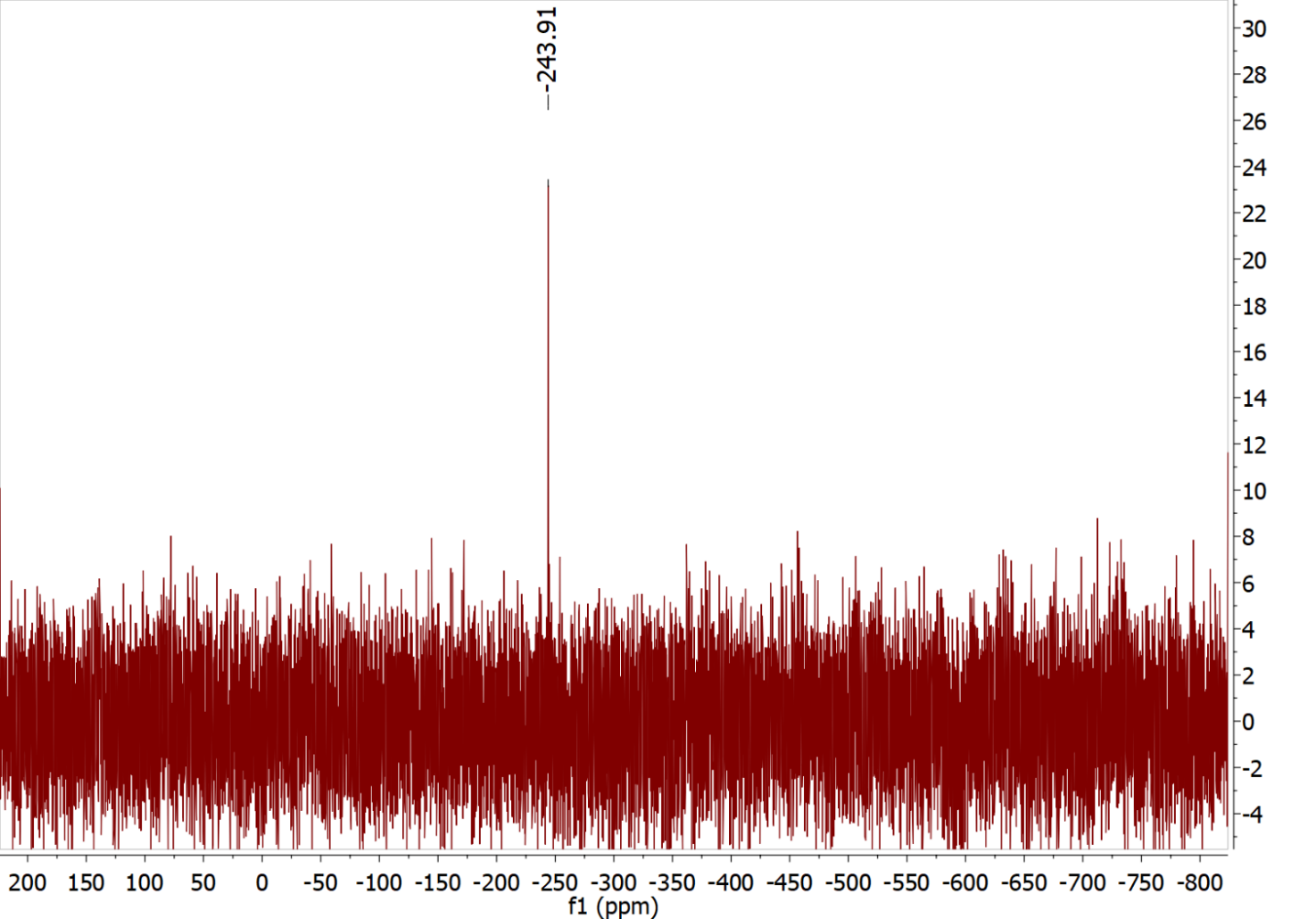


**S28.** ^119^Sn{^1^H} NMR spectrum of complex **2** in DMSO-d_6_.


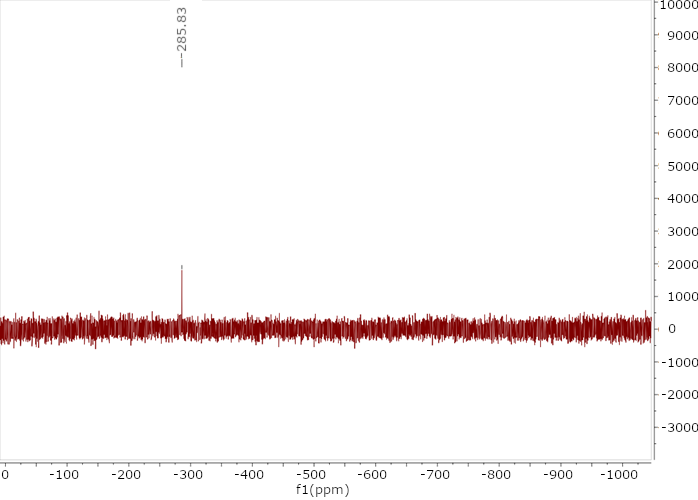


**S29.** ^119^Sn{^1^H} NMR spectrum of complex **3** in DMSO-d_6_.


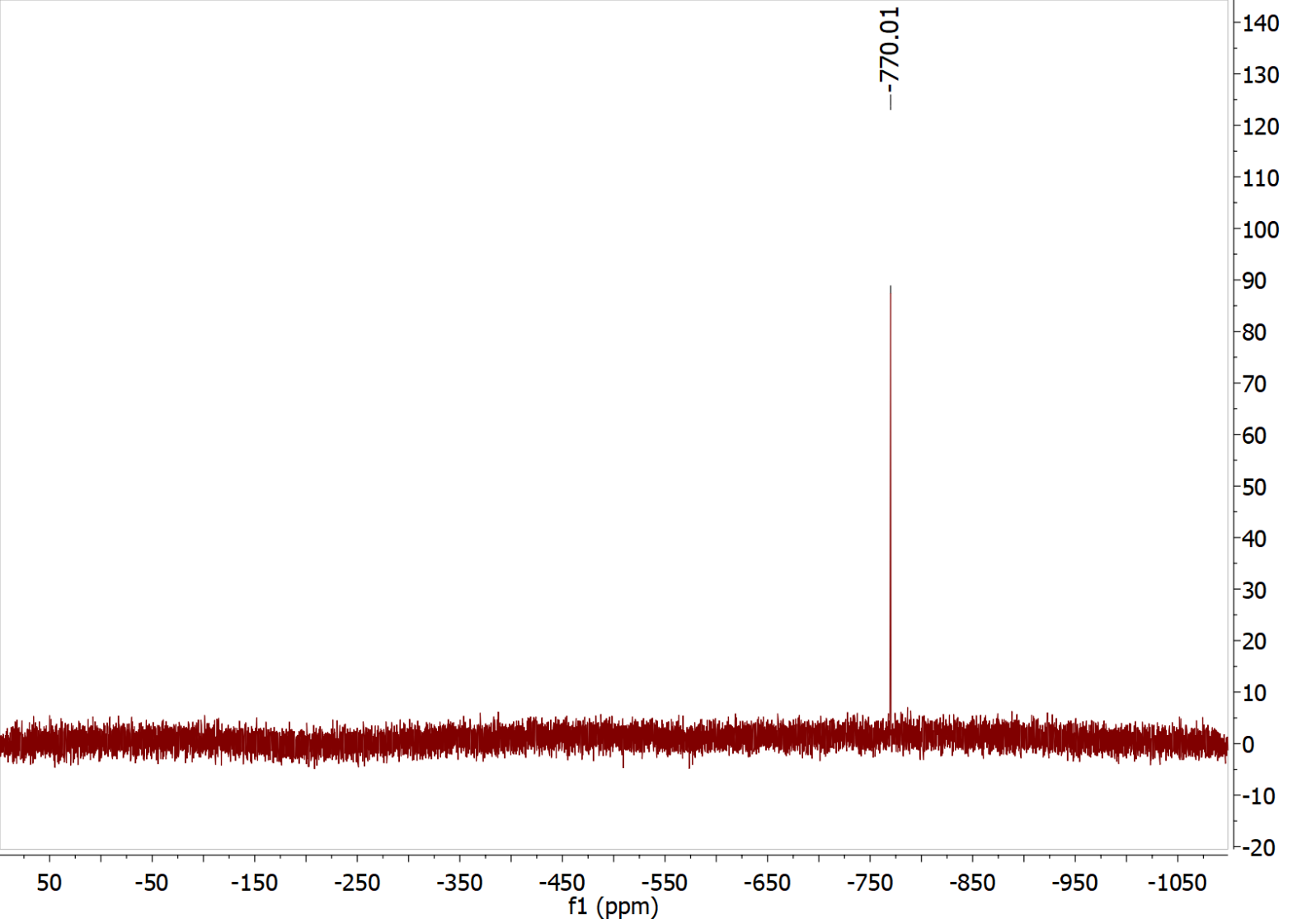
**S30.** ^119^Sn{^1^H} NMR spectrum of complex **4** in DMSO-d_6_.


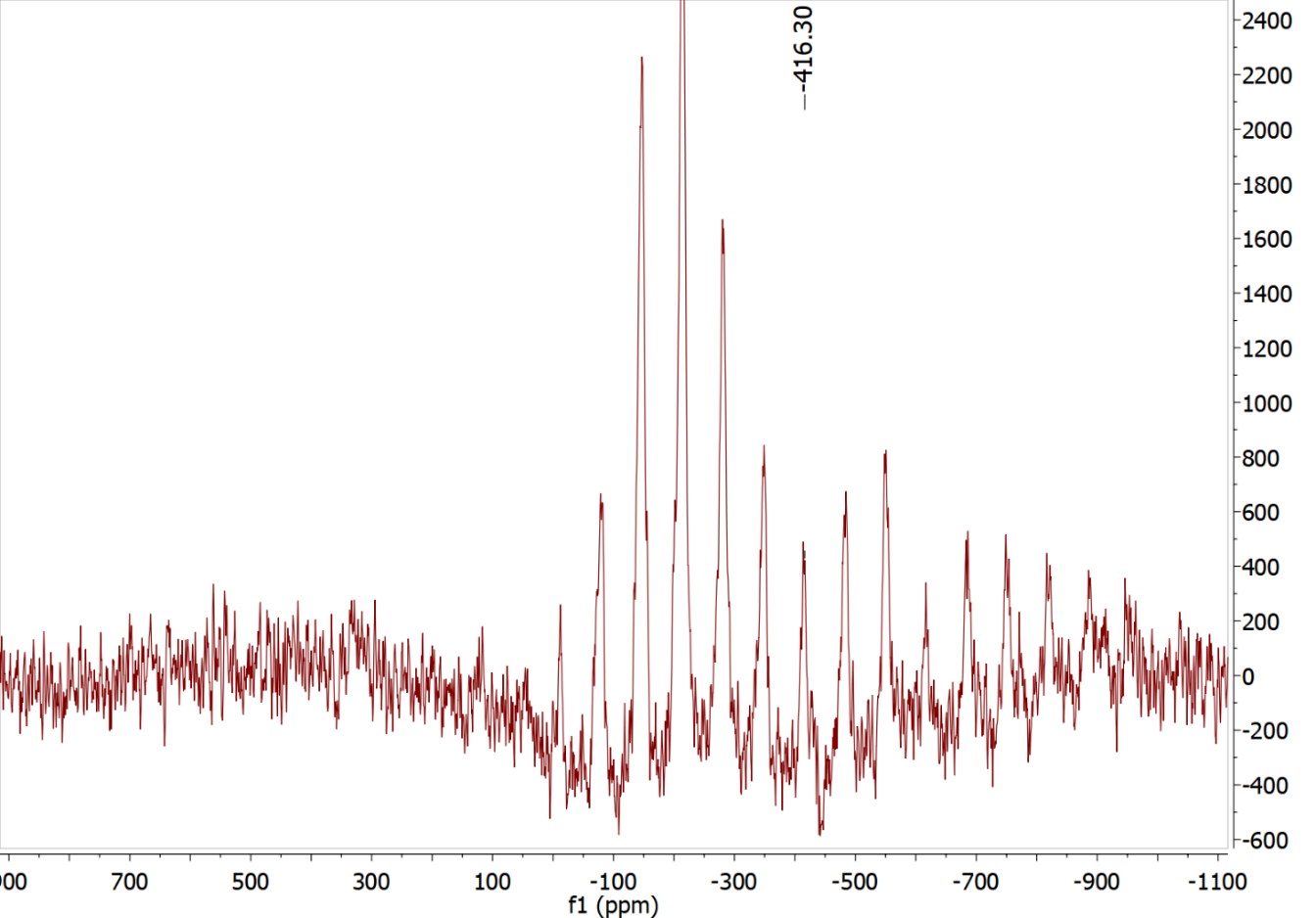


**S31.** ^119^Sn{^1^H} CP/MAS NMR spectrum of complex **1**

**S32.** ^119^Sn{^1^H} CP/MAS NMR spectrum of complex **2**


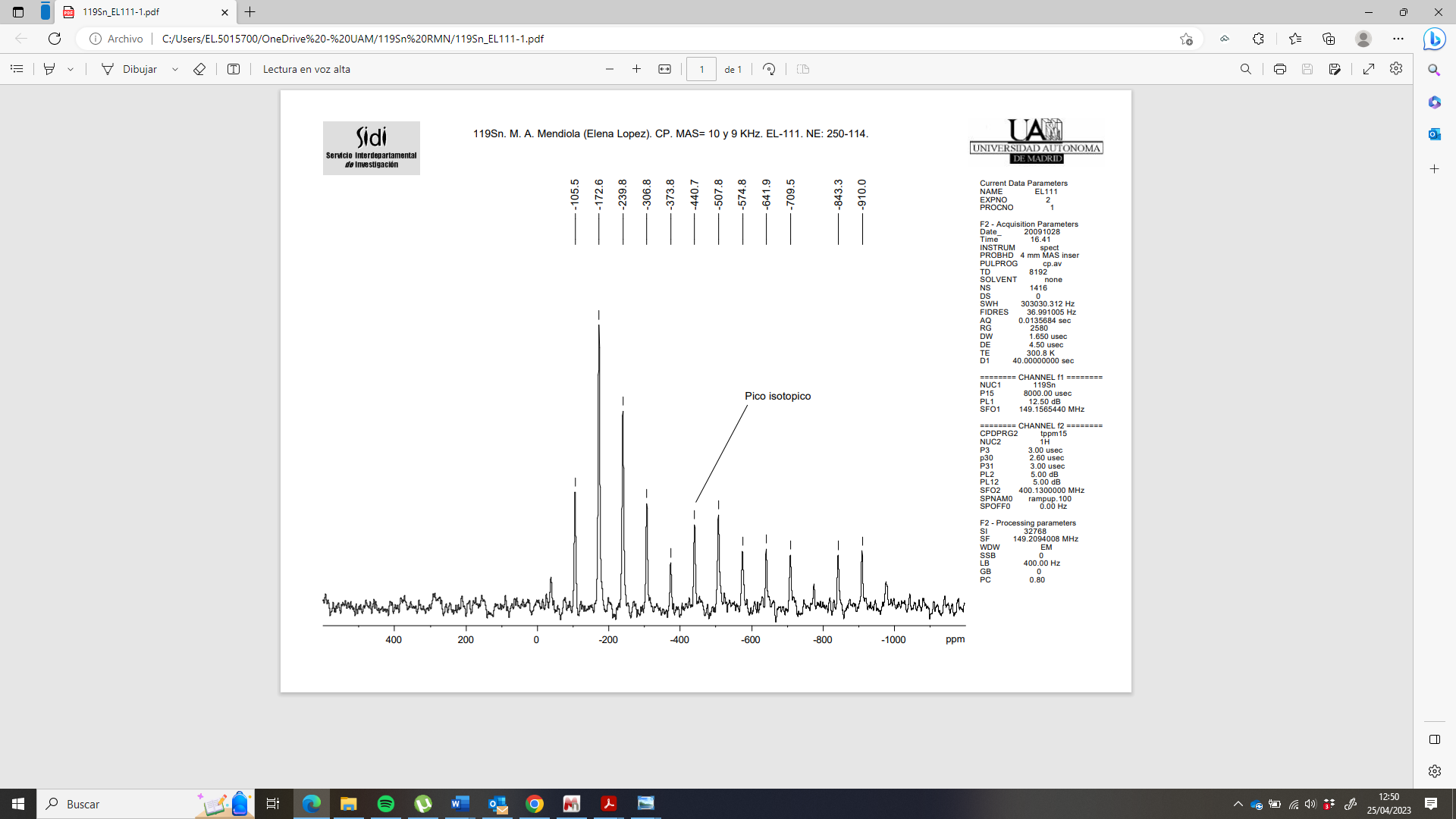


Isotropic peak


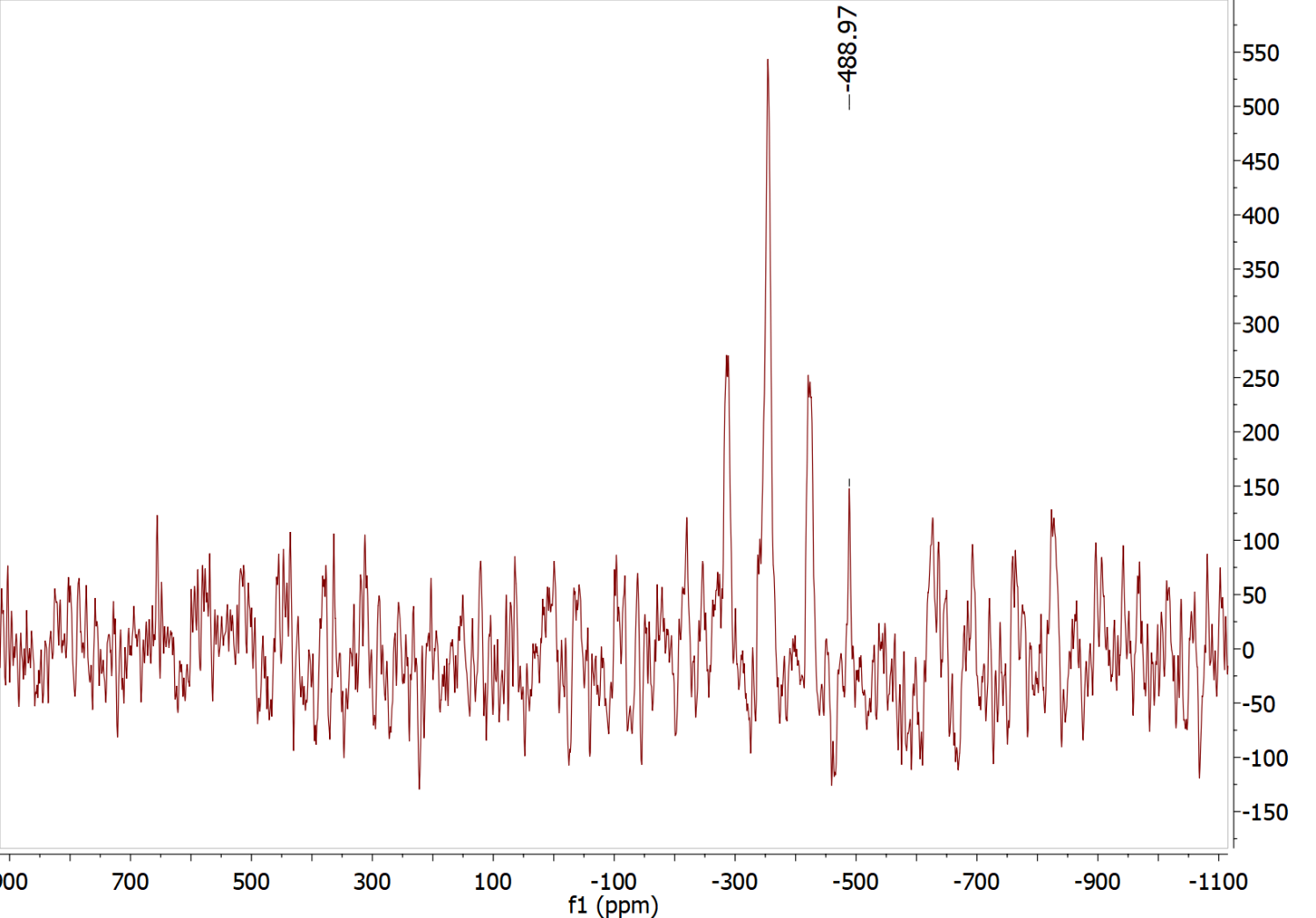


**S33.** ^119^Sn{^1^H} CP/MAS NMR spectrum of complex **3**


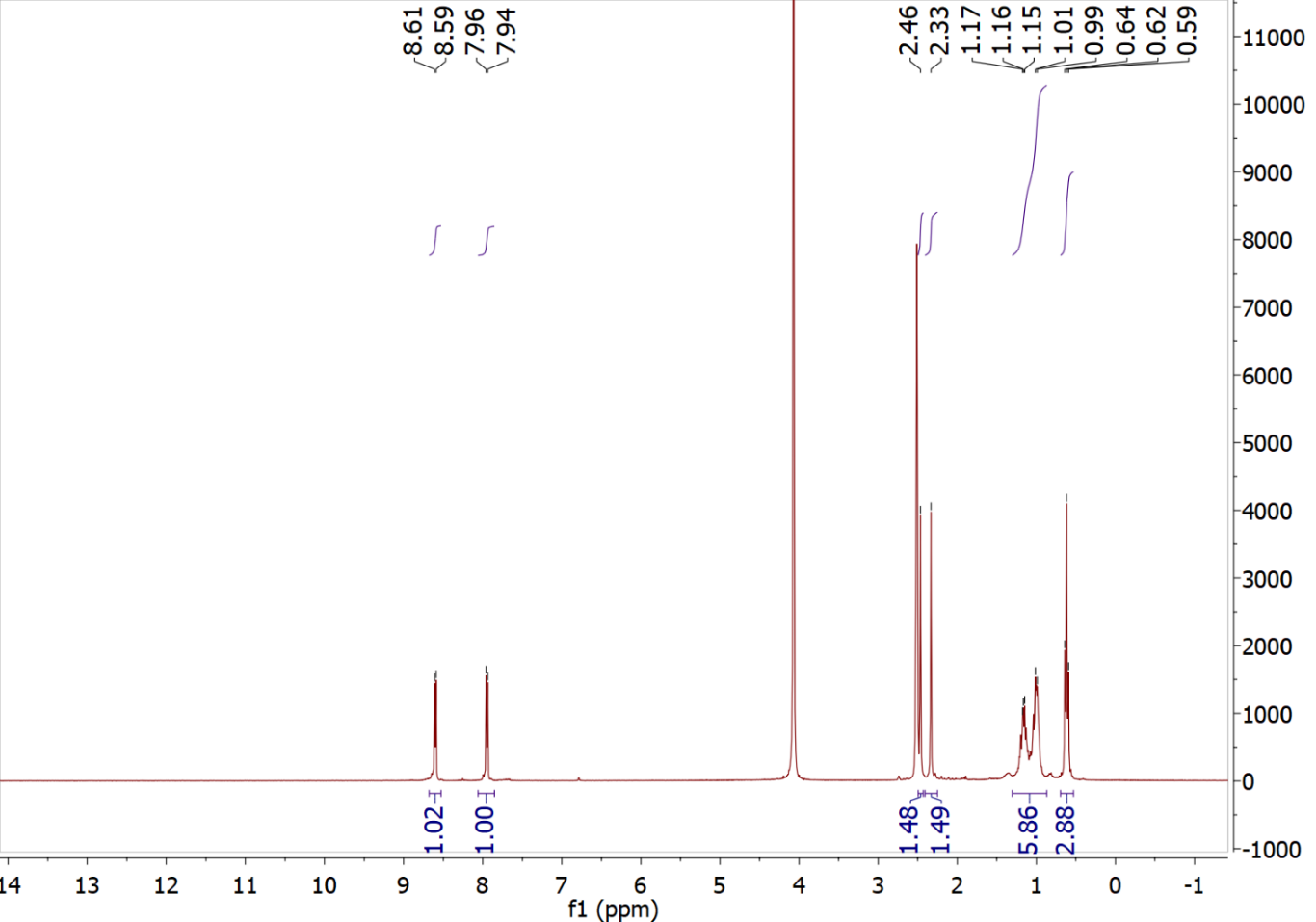

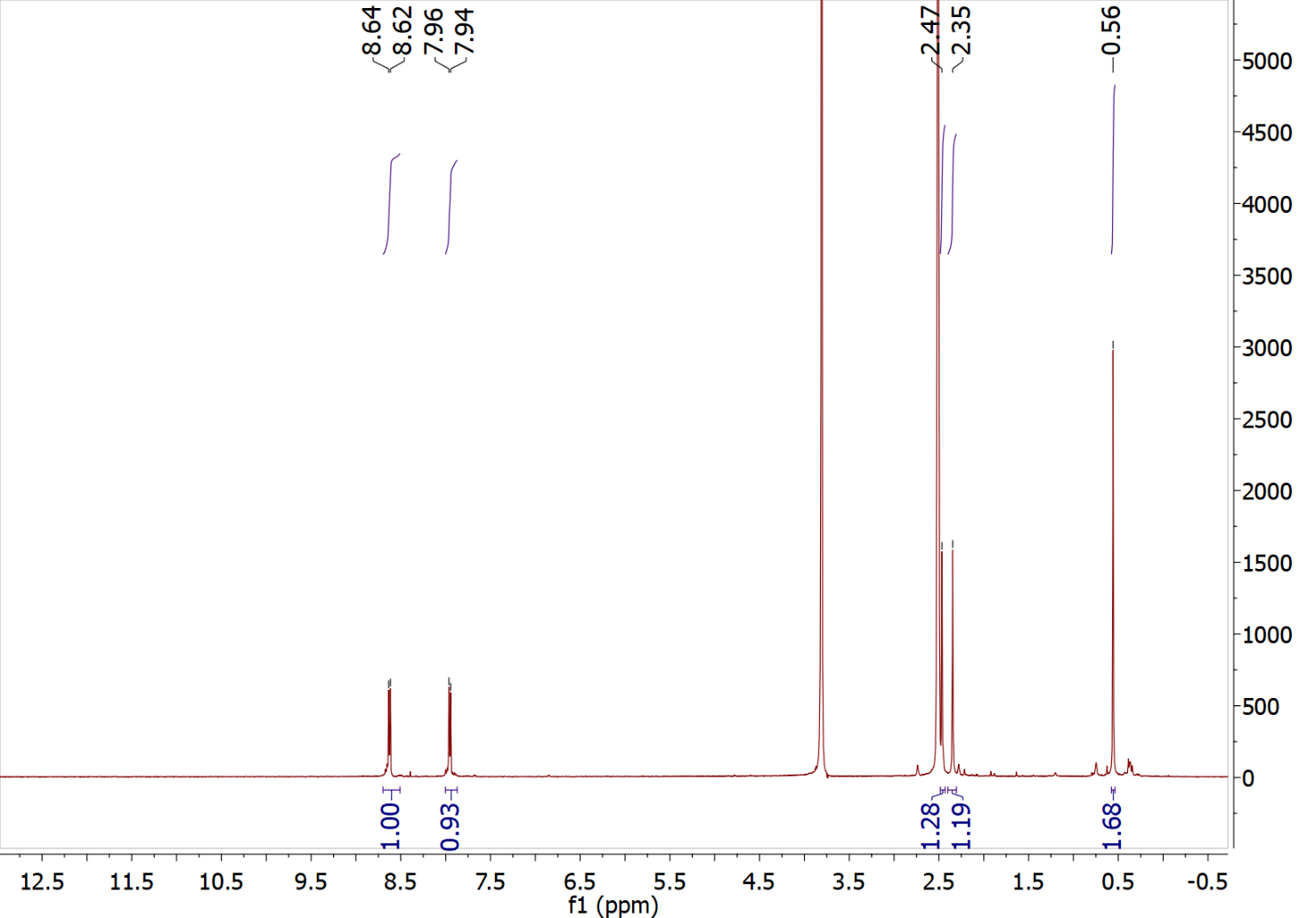
**S34.** ^1^H NMR spectrum of complex **1** in DMSO-d_6_+D_2_O 1:2.

**S35.** ^1^H NMR spectrum of complex **2** in DMSO-d_6_+D_2_O 1:2.


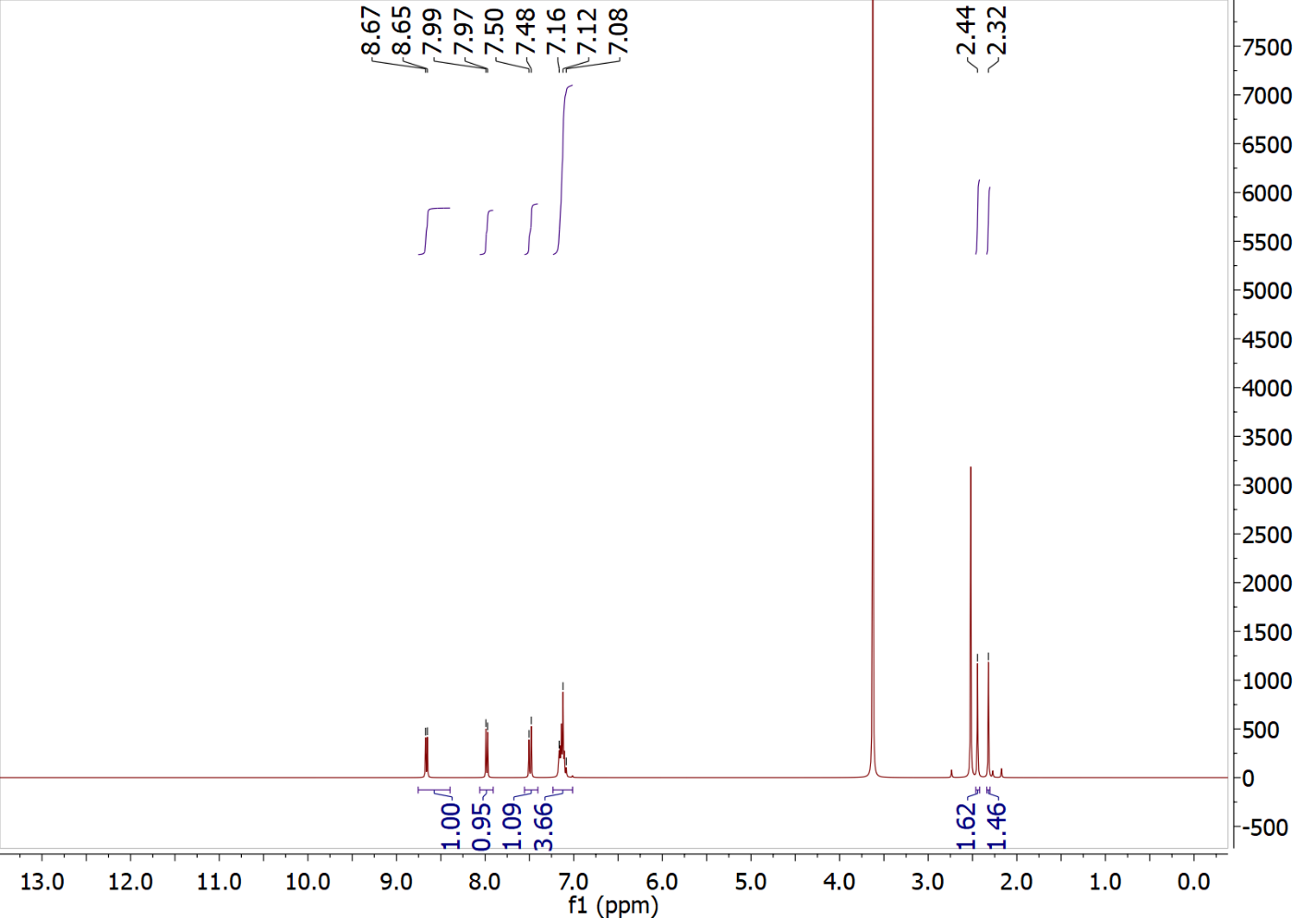


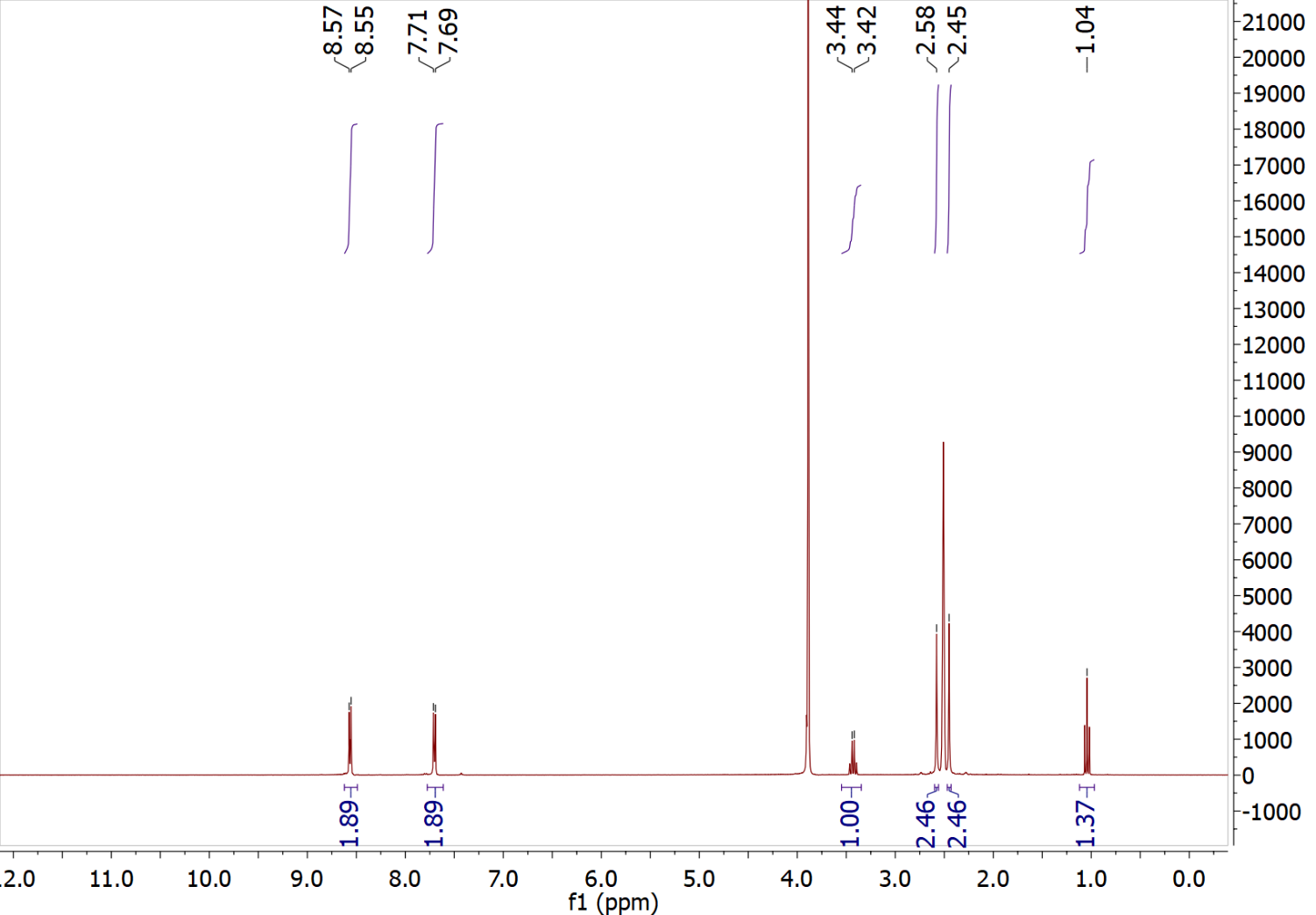
**S36.** ^1^H NMR spectrum of complex **3** in DMSO-d_6_+D_2_O 1:2.

**S37.** ^1^H NMR spectrum of complex **4** in DMSO-d_6_+D_2_O 1:2.

**S38.** Percentage of cell viability upon treatment with different concentrations of compound 3.

| Percentage of cell viability upon treatment with compound 3 | | | | | |
| --- | --- | --- | --- | --- | --- |
| Concentration  (µM) | Hela | MDA-MB 231 | HepG2 | PC3 | WI-38 |
| 0 | 100 | 100 | 100 | 100 | 100 |
| 0.25 | 70 | 65.43 | 80.36 | 82.17 | 98.36 |
| 0.5 | 46.24 | 50.06 | 70.36 | 76.09 | 95.32 |
| 1 | 30.21 | 28.63 | 58.39 | 60.16 | 80.17 |
| 2 | 25.19 | 20.19 | 45.27 | 48.24 | 76.21 |
| 5 | 20.16 | 18.37 | 30.68 | 35.73 | 70.24 |

~~
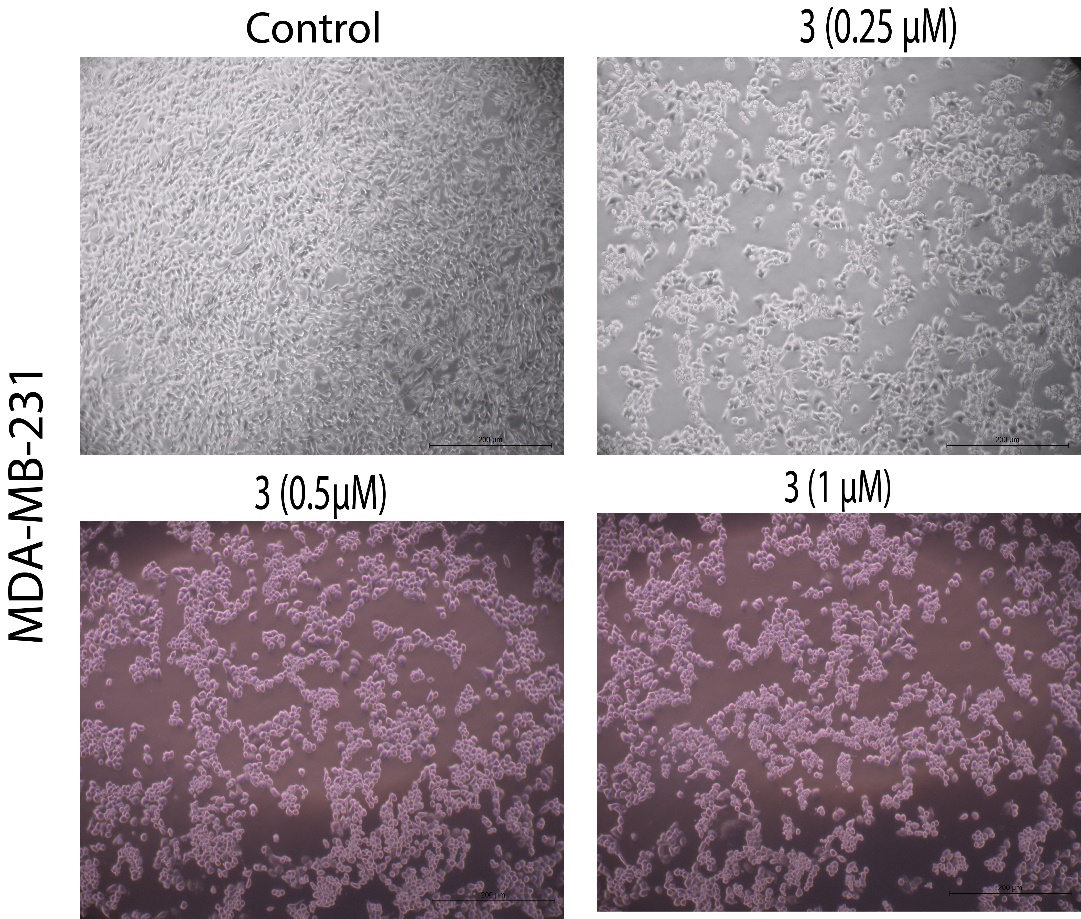
~~

**S39.** Microscopic images (20×) of MDA-MB-231 cells after treatment with different concentrations of complex **3** showing cytotoxic as well as cytostatic effect. Primarily MDA-MB 231 cells were seeded at a density of 1×10^6^ cells/mL in each 35 mm Petri dish for 24 h. Thereafter, the cells were treated with increasing concentrations of complex **3** (0.25 µM, 0.5 µM and 1µM) for 24 h.
